# Inhibition of the long non‐coding RNA ZFAS1 attenuates ferroptosis by sponging miR‐150‐5p and activates CCND2 against diabetic cardiomyopathy

**DOI:** 10.1111/jcmm.16890

**Published:** 2021-10-05

**Authors:** Tingjuan Ni, Xingxiao Huang, Sunlei Pan, Zhongqiu Lu

**Affiliations:** ^1^ Department of Emergency Intensive Care Unit the First Affiliated Hospital Wenzhou Medical University Wenzhou Zhejiang China; ^2^ Department of Cardiology Zhejiang University Hangzhou Zhejiang China; ^3^ Department of Coronary Care Unit the First Affiliated Hospital Wenzhou Medical University Wenzhou Zhejiang China

**Keywords:** CCND2, diabetic cardiomyopathy, ferroptosis, lncRNA‐ZFAS1, miR‐150‐5p

## Abstract

Diabetic cardiomyopathy (DbCM) is responsible for increased morbidity and mortality in patients with diabetes and heart failure. However, the pathogenesis of DbCM has not yet been identified. Here, we investigated the important role of lncRNA‐ZFAS1 in the pathological process of DbCM, which is associated with ferroptosis. Microarray data analysis of DbCM in patients or mouse models from GEO revealed the significance of ZFAS1 and the significant downregulation of miR‐150‐5p and CCND2. Briefly, DbCM was established in high glucose (HG)–treated cardiomyocytes and db/db mice to form in vitro and in vivo models. Ad‐ZFAS1, Ad‐sh‐ZFAS1, mimic miR‐150‐5p, Ad‐CCND2 and Ad‐sh‐CCND2 were intracoronarily administered to the mouse model or transfected into HG‐treated cardiomyocytes to determine whether ZFAS1 regulates miR‐150‐5p and CCND2 in ferroptosis. The effect of ZFAS1 on the left ventricular myocardial tissues of db/db mice and HG‐treated cardiomyocytes, ferroptosis and apoptosis was determined by Masson staining, immunohistochemical staining, Western blotting, monobromobimane staining, immunofluorescence staining and JC‐1 staining. The relationships among ZFAS1, miR‐150‐5p and CCND2 were evaluated using dual‐luciferase reporter assays and RNA pull‐down assays. Inhibition of ZFAS1 led to reduced collagen deposition, decreased cardiomyocyte apoptosis and ferroptosis, and attenuated DbCM progression. ZFAS1 sponges miR‐150‐5p to downregulate CCND2 expression. Ad‐sh‐ZFAS1, miR‐150‐5p mimic, and Ad‐CCND2 transfection attenuated ferroptosis and DbCM development both in vitro and in vivo. However, transfection with Ad‐ZFAS1 could reverse the positive effects of miR‐150‐5p mimic and Ad‐CCND2 in vitro and in vivo. lncRNA‐ZFAS1 acted as a ceRNA to sponge miR‐150‐5p and downregulate CCND2 to promote cardiomyocyte ferroptosis and DbCM development. Thus, ZFAS1 inhibition could be a promising therapeutic target for the treatment and prevention of DbCM.

**Abbreviations**
4‐HNE: 4‐hydroxynonenal
ceRNAs: competing endogenous RNAs
DbCM: diabetic cardiomyopathy
FTH1: ferritin heavy chain
GEO: Gene Expression Omnibus
GPX4: glutathione peroxidase 4
GSH: glutathione
HG: high glucose
lncRNAs: long non‐coding ribonucleic acids
MBB: monobromobimane
miRNAs: micro‐RNAs
ZFAS1: zinc finger antisense 1

## 1 INTRODUCTION

Patients with diabetes are vulnerable to a series of cardiovascular complications, one of the most serious complications associated with heart failure (HF).^1^, ^2^ As the incidence of diabetes (expected to reach 693 million by 2045) increases,^3^ HF due to diabetes has become a worldwide epidemic.^4^, ^5^ In the clinical setting, diabetes is present in one third of all HF patients. Further, diabetes has been identified as an independent predictor of adverse outcomes.^6^ According to a report published five decades ago by the Framingham Heart Study, a fourfold to fivefold increase in the risk of HF has been identified among patients with diabetes,^2^, ^7^, ^8^ where diabetic cardiomyopathy (DbCM) is recognized as a proximate cause. Despite extensive research on DbCM,^9^ the full spectrum of its pathogenesis and its relative contribution to the HF phenotype in diabetes has not been revealed.

Long non‐coding RNAs (lncRNAs), which are non‐coding RNAs exceeding 200 nucleotides in length, participate in multiple biological processes, including cell metabolism, cell proliferation, cell fate determination and apoptosis, ultimately resulting in several pathological conditions, such as cancer and Alzheimer's disease. Emerging evidence has shown that lncRNAs regulate cardiac diseases,^10^, ^11^, ^12^, ^13^ such as cardiac‐related lncRNA zinc finger antisense 1 (ZFAS1) being associated with acute myocardial infarction (MI).^14^, ^15^, ^16^, ^17^ Nevertheless, the mechanism underlying the role of lncRNA‐ZFAS1 in DbCM has not been identified.

lncRNAs have been shown to act as competing endogenous RNAs (ceRNAs) to sponge macromolecules, such as micro‐RNAs (miRNAs) and proteins,^18^ which are associated with a wide range of physiological, biological and pathological processes, including those in the case of cardiac diseases.^19^, ^20^ A previous clinical study revealed that miR‐150‐5p levels were significantly reduced in patients with HF. Thus, miR‐150‐5p was identified as an independent predictor of HF.^21^ The miR‐150‐5p gene was found to mitigate apoptosis in sepsis‐induced myocardial depression,^22^ alleviate the progression of myocardial fibrosis^23^ and rescue cardiomyocytes from hypoxia‐induced injury under the command of the lncRNA, FOXD3‐AS1.^24^ However, the role of miR‐150‐5p in DbCM and its relationship with lncRNA‐ZFAS1 have not been studied. Cyclin D2 (CCND2) regulates the proliferation of cardiac myocytes,^25^ which is beneficial for cardiac dysfunction,^26^ and activates cell cycle progression to enhance myocardial repair.^27^ The role of CCND2 in DbCM has also not yet been studied.

Ferroptosis, an iron‐dependent regulated necrosis associated with a new form of regulatory cell death, was first described in 2012.^28^ Ferroptosis can induce the pathological processes of cancer, stroke, cardiovascular disease and kidney failure.^29^, ^30^ Glutathione peroxidase 4 (GPX4) can terminate ferroptosis, which occurs in caspase and necrosomal complexes.^31^ A recent report demonstrated that inhibiting ferroptosis could decrease mitochondrial iron to alleviate DOX‐induced cardiac injury^32^; however, its role in DbCM has not been explored.

In this study, HG‐treated cardiomyocytes and db/db mice were used to simulate DbCM in vitro and in vivo, and lncRNA‐ZFAS1 was assessed to demonstrate that ZFAS1 inhibition alleviates the development of DbCM by reducing ferroptosis by stabilizing miR‐150‐5p to activate CCND2.

## 2 MATERIALS AND METHODS

### 2.1 Ethics and animal experiments

Animal experiments were performed in accordance with the Institutes of the First Affiliated Hospital of Wenzhou Medical University Health Guidelines on the Use of Laboratory Animals to ensure the humanitarian care of the experimental animals.

**FIGURE 1:**
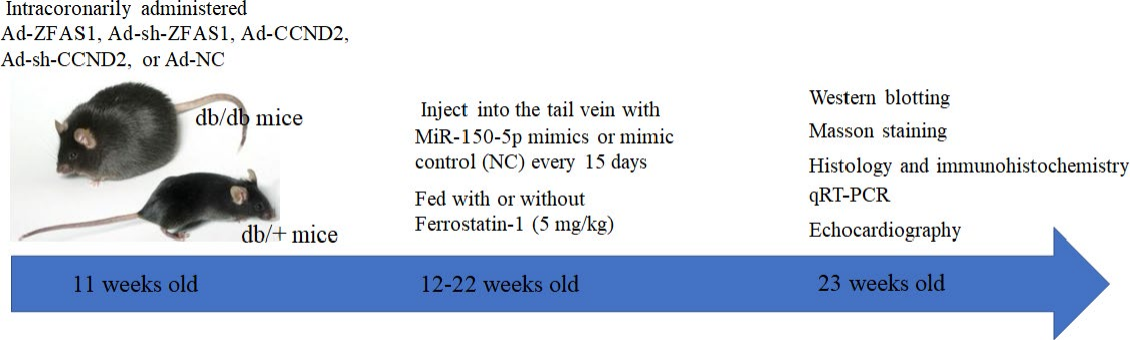
Schematic details on a temporal scale about the animal experiments.

To simulate the animal model of diabetic cardiomyopathy, male db/+ mice and db/db mice (age, 7 weeks, weight, 24 g) were fed a normal diet for 4 weeks and kept at 24℃ under a 14‐h light/8‐h dark cycle. The animals were purchased from the Model Animal Research Center of Nanjing University (Nanjing, China). Diabetic mice were intracoronarily administered equal volumes (80 μl) of adenoviruses Ad‐ZFAS1, Ad‐sh‐ZFAS1, Ad‐CCND2, Ad‐sh‐CCND2 or Ad‐NC.^33^ miR‐150‐5p mimics and mimic control (NC) were injected into the tail vein of mice (50 μg/kg) every 15 days for 12 weeks. Db/db mice were treated with or without ferrostatin‐1 (Fer‐1, ferroptosis inhibitor; Sigma‐Aldrich, 5 mg/kg) for an additional 12 weeks (Figure 1). Each group consisted of eight mice.

### 2.2 Primary culture of neonatal cardiomyocytes and cell transfection

To obtain primary cardiomyocytes, the cardiac tissues of newborn (1–2 days old) mice were isolated as in the previous article.^34^ Cardiomyocytes were transfected with adenoviruses Ad‐ZFAS1, Ad‐sh‐ZFAS1, Ad‐CCND2 and Ad‐sh‐CCND2 (10 μl/ml, MOI: 100:1, the titre of the adenoviruses was approximately 1.2×1010 PFU/mL; Hanbio Technology Ltd., Shanghai, China) in serum‐free Dulbecco's modified Eagle's medium for 6–8 h, and miR‐150‐5p mimics and mimic control (NC) (Sigma, St. Louis, MO, USA) were transfected into cardiomyocytes using Lipofectamine® 3000 (Invitrogen; Carlsbad, CA, USA) and cultured in the absence or presence of high glucose levels (HG, 25 mmol/L glucose) exposed with the same osmolality with or without 0.4 μM Fer‐1 (Sigma‐Aldrich).

### 2.3 Microarray‐based gene expression data analysis

To obtain differential genetic analyses of diabetes, the microarray data of patients with and without diabetes experiencing HF based on the GSE26887 data set, the micro‐RNAs involved in the pathophysiology of DbCM based on the GSE44179 data set, and expression data using rat ventricles on days 3, 28 and 42 after the STZ injection based on the GSE4745 data set were obtained from the Gene Expression Omnibus (GEO) database (https://www.ncbi.nlm.nih.gov/geo/). DbCM‐related genes were screened using Excel (Microsoft), |log2FC| > 2.0 and adj. *p*. Val (*p* value after correction) <0.05.

### 2.4 Dual‐luciferase reporter assay

The dual‐luciferase reporter assay and the online database starBase v2.0 were employed to predict the binding site of miR‐150‐5p with lncRNA‐ZFAS1 (http://starbase.sysu.edu.cn/agoClipRNA.php?source=lncRNA). The online database, TargetScan (http://www.targetscan.org/mamm_31/), was used to predict whether miR‐150‐5p could bind to CCND2. Wild‐type (wt‐ZFAS1 and wt‐CCND2) and mutant (mut‐ZFAS1 and mut‐CCND2) sequences were designed and synthesized according to the predicted binding sites. Sequences were inserted into the luciferase reporter vector (PGL3‐Basic). The human embryonic kidney cell line HEK293T cells (ATCC, Manassas, VA) were cultured in 24‐well plates and allowed to reach 80% confluence. Thereafter, the cells were co‐transfected with the miR‐150‐5p mimic (30 n M) or miR‐138‐5p mimic negative controls (30 nM; GenePharma, Shanghai, China) using the Lipofectamine 3000 reagent (Thermo Fisher Scientific, USA). After 48 h, luciferase activity was detected using the Dual‐Luciferase Reporter Assay Kit (Promega, USA).

### 2.5 Quantitative reverse‐transcription‐polymerase chain reaction (qRT‐PCR)

The expression levels of ZFAS1, CCND2 and miR‐150‐5p were determined using qRT‐PCR. Total RNA from the left ventricular tissue and cardiomyocytes was extracted using the TRIzol reagent (Invitrogen), first‐strand complementary DNA was extracted using the PrimeScript RT Reagent Kit with gDNA Eraser and TB Green Premix Ex Tag II (TaKaRa Bio, Japan), and miRNA was extracted from total RNA using the Mir‐X miRNA First‐Strand Synthesis Kit and Mir‐X miRNA qRT‐PCR TB Green Kit (TaKaRa Bio, Japan). β‐Actin was used as an internal control in the determination of ZFAS1 and CCND2 expression, whereas U6 was used in the case of miR‐150‐5p expression. qRT‐PCR was performed according to the manufacturer's protocol using a QuantStudio 5 Real‐Time PCR System (Thermo Fisher Scientific, USA). The following primer sequences were employed: ZFAS1 (forward: 5′‐ACGTGCAGACATCTACAAC CT‐3′ and reverse: 5′‐TACTTCCAACACCCGCAT‐3′); miR‐150‐5p (forward: 5′‐TCGG CGTC TCCC AACC CTTG TAC‐3′ and reverse: 5′‐GTCG TATC CAGT GCAG GGTC CGAG GT‐3′); and CCND2 (forward: 5′‐AGAGCCACCGGTATGGAGCTGCTGTGCCACGAGGT‐3′ and reverse: 5′‐CTGCAGGCGCGCCGAATTTTTTTTTTAAGTTTCACCCT‐3′).

### 2.6 RNA pull‐down assay

To detect whether ZFAS1 can bind to miR‐150‐5p, biotinylated wild‐type miR‐150‐5p (Bio‐wt‐150‐5p), biotinylated mutant miR‐150‐5p (Bio‐mut‐150‐5p) or biotinylated miRNA that was not complementary to ZFAS1 (Bio‐NC) was transfected into primary cardiomyocytes. Forty‐eight hours after transfection, cardiomyocytes were retrieved for the biotin‐based pull‐down assay (Thermo Fisher Scientific, USA), which was performed according to the manufacturer's protocol. The expression levels of ZFAS1 were measured using real‐time PCR.

Magnetic beads coated with a ZFAS1 probe or a random probe were added to the cardiomyocyte lysate. After washing and enrichment of the bead/RNA complex, miR‐150‐5p was eluted from streptavidin beads. The expression level of miR‐150‐5p was determined through Northern blotting (Thermo Fisher Scientific, USA), according to the manufacturer's protocol.

### 2.7 Immunofluorescence staining

Protein localization of the ferritin heavy chain was determined by immunohistochemical staining. After fixing with 4% paraformaldehyde for 15 min, permeabilization with 0.5% Triton X‐100 for 20 min and blocking with 4% goat serum for 30 min at 37℃, adherent experimental cardiomyocytes were incubated with the primary antibody against ferritin heavy chain (FTH1, ab65080) at 4℃ overnight. Following incubation with DyLight 488 and 594 AffiniPure Goat IgG (H+L) for 1 h at 37℃ and counterstaining with 0.1 μg/ml DAPI (P36941; Invitrogen) for 3 min, images were captured using a Nikon Eclipse Ti‐U fluorescence microscope.

### 2.8 Histology and immunohistochemistry

The relative expression of FTH1 and 4‐HNE was determined through immunohistochemical staining of the left ventricular myocardial tissues of experimental mice. After dewaxing in a 60℃ incubator, hydration with xylene and anhydrous ethanol, and antigen repair with citrate antigen retrieval solution (Beyotime, China), 5‐mm‐thick sections of the left ventricular myocardial tissues were incubated with primary antibodies against ferritin heavy chain (FTH1, ab65080) and 4‐hydroxynonenal (4‐HNE, ab46545) at 4℃ overnight. Sections were then incubated with secondary antibodies at 37℃ for 30 min and stained with 3,3'‐diaminobenzidine (Gene Tech, China) at 37℃ for 5 min. Images were then immediately captured in the dark using a Nikon Eclipse Ti‐U fluorescence microscope (Tokyo, Japan).

### 2.9 Masson staining

Cardiac collagen content was measured with Masson staining. After dewaxing in a 60℃ incubator, hydrated with xylene and anhydrous ethanol, and stained with haematoxylin and Lichun red acid, 5‐mm‐thick sections of the left ventricular myocardial tissues were finally stained with 1% phosphomolybdic acid. Images were immediately captured in the dark using a Nikon Eclipse Ti‐U fluorescence microscope (Tokyo, Japan). Collagen fibres were blue (aniline blue) or green (bright green), and the muscle fibres and cellulose were red.

### 2.10 Monobromobimane (MBB) staining

MBB staining was used to determine glutathione (GSH) levels in cardiomyocytes. Briefly, cardiomyocytes were stained with MBB (20 μM; Sigma‐Aldrich, USA) in PBS for 15 min at 37℃, and images were immediately captured in the dark using a Nikon Eclipse Ti‐U fluorescence microscope (Tokyo, Japan).

### 2.11 JC‐1 staining

JC‐1 staining was used to determine the mitochondrial membrane potential. Cardiomyocytes were stained with JC‐1 (MCE, NJ, USA) in PBS for 30 min at 37℃. Thereafter, the cells were immediately imaged in the dark using a Nikon Eclipse Ti‐U fluorescence microscope (Tokyo, Japan).

### 2.12 Western blotting

The relative protein expression of GPX4, CCND2, cleaved caspase‐3, miR‐150‐5p, Bcl‐2, Bax and β‐actin was determined using Western blotting. The left ventricular tissue and cardiomyocytes were lysed for protein retrieval. The proteins were then separated via sodium dodecyl sulphate‐polyacrylamide gel electrophoresis and transferred onto 0.45‐μm polyvinylidene difluoride transfer membranes (PVDF; Millipore, USA). After blocking and incubation with the primary antibodies, including GPX4 (ab125066), CCND2 (ab207604), cleaved caspase 3 (ab13847), Bcl‐2 (ab32124), Bax (ab53154) and β‐actin (ab8226) overnight at 4℃, the PVDF membranes were incubated with peroxidase‐conjugated secondary antibodies, including anti‐mouse and anti‐rabbit antibodies (Abbkine, Redlands, CA). Visualization was then performed with a ECL Plus Detection Reagent (Sigma, USA).

### 2.13 Echocardiography

Cardiac function was evaluated using echocardiography. We used a parasternal long‐axis view. Transthoracic echocardiography was performed using a Philips iE33 system (Philips Medical, the Netherlands). The left ventricular anteroposterior diameter was measured using the parasternal long‐axis view. The left and right ventricular diameters and superior and inferior diameters were measured in the apical 4‐chamber view, and left ventricular end‐systolic diameter (LVESD) and left ventricular end‐diastolic diameter (LVEDD) were recorded. Next, left ventricular fraction shortening (LVFS) and the left ventricular ejection fraction (LVEF) were calculated using a computer algorithm after three successive cardiac cycles. Early‐diastolic peak mitral valve flow velocity and atrial systolic velocity were recorded using the pulsed Doppler technique, and the E/A ratio was calculated to reflect left ventricular diastolic function. Each group consisted of eight mice.

### 2.14 Statistical analysis

SPSS Statistics version 26.0 (SPSS Inc., Chicago, IL, USA) was used for data analysis. Data are expressed as mean ± standard deviation. Student's *t* test and analysis of variance were used to analyse the differences between two or more groups. All experiments were repeated at least three times. Statistical significance was set at *p* < 0.05.

## 3 RESULTS

### 3.1 Upregulated ZFAS1 expression and increased ferroptosis in mice with DbCM‐ and HG‐treated cardiomyocytes

**FIGURE 2:**
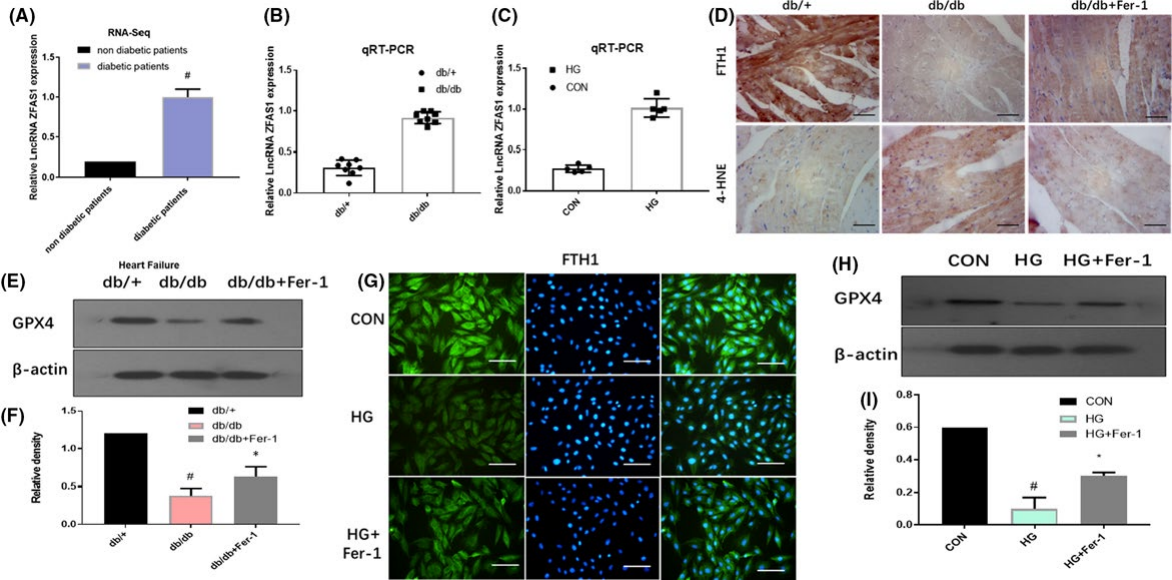
ZFAS1 is involved in response to diabetic cardiomyopathy– and high glucose–induced ferroptosis. (A) ZFAS1 expression levels in cardiomyocytes of patients with and without diabetes based on RNA‐sequencing data (GSE26887). (B) RT‐qPCR analysis of ZFAS1 expression in the left ventricular myocardial tissues of db/+ and db/db mice. (C) RT‐qPCR analysis of ZFAS1 expression in control cardiomyocytes and HG‐treated cardiomyocytes. (D) Relative expression of FTH1 and 4‐HNE in the left ventricular myocardial tissues of db/+ and db/db mice treated with or without the Fer‐1 was determined by immunohistochemical staining. (E‐F) Relative protein expression level of GPX4 in the left ventricular myocardial tissues of db/+ and db/db mice treated with or without the Fer‐1 was assessed by Western blot analysis. (G) Immunofluorescence against FTH1 (green) in control cardiomyocytes and HG‐treated cardiomyocytes with or without Fer‐1. (H–I) Relative protein expression level of GPX4 in control cardiomyocytes and HG‐treated cardiomyocytes with or without Fer‐1 was assessed by Western blotting. #*p* < 0.05 versus CON group or db/+ group; data are expressed as mean ±standard deviation (SD, *n* = 3).

The differential HF‐related gene profiles of patients with and without diabetes were screened using the GSE26887 data set from the GEO database. lncRNA‐ZFAS1 was found to be significantly upregulated in diabetic patients with HF (Figure 2A). Therefore, to determine whether ZFAS1 is involved in DbCM, the expression level of ZFAS1 in the left ventricular myocardial tissues of db/db mice and HG‐treated cardiomyocytes was determined by qRT‐PCR. ZFAS1 was significantly upregulated in the same manner (Figure 2B, C).

FTH1, a key iron storage protein involved in iron metabolism that acts as a ferritinophagy biomarker, was decreased in the left ventricular myocardial tissues of db/db mice, which was alleviated by treatment with ferrostatin‐1(Fer‐1), a ferroptosis inhibitor, as evaluated by immunohistochemical staining (Figure 2D). Furthermore, 4‐HNE, the final product of lipid hydroperoxidation, was increased in the left ventricular myocardial tissues of db/db mice; however, it was downregulated in the Fer‐1 + db/db group, as demonstrated by immunohistochemical staining (Figure 2D). Western blotting showed that GPX4, which could terminate the process of ferroptosis, was decreased in the left ventricular myocardial tissues of db/db mice; however, it was upregulated in the Fer‐1 + db/db group (Figure 2E‐F). Consistent with the in vivo results, FTH1 was rarely observed in the cytoplasm of HG‐treated cardiomyocytes (Figure 2G). Furthermore, the expression level of GPX4 was reduced in HG‐treated cardiomyocytes; however, it was upregulated in the Fer‐1 + HG group, as measured by Western blotting (Figure 2H–I). Collectively, these findings indicate that the expression level of ZFAS1 is upregulated and ferroptosis is increased in DbCM‐ and HG‐treated cardiomyocytes.

### 3.2 Inhibition of ZFAS1 repressed ferroptosis in mice with DbCM‐ and HG‐treated cardiomyocytes

**FIGURE 3:**
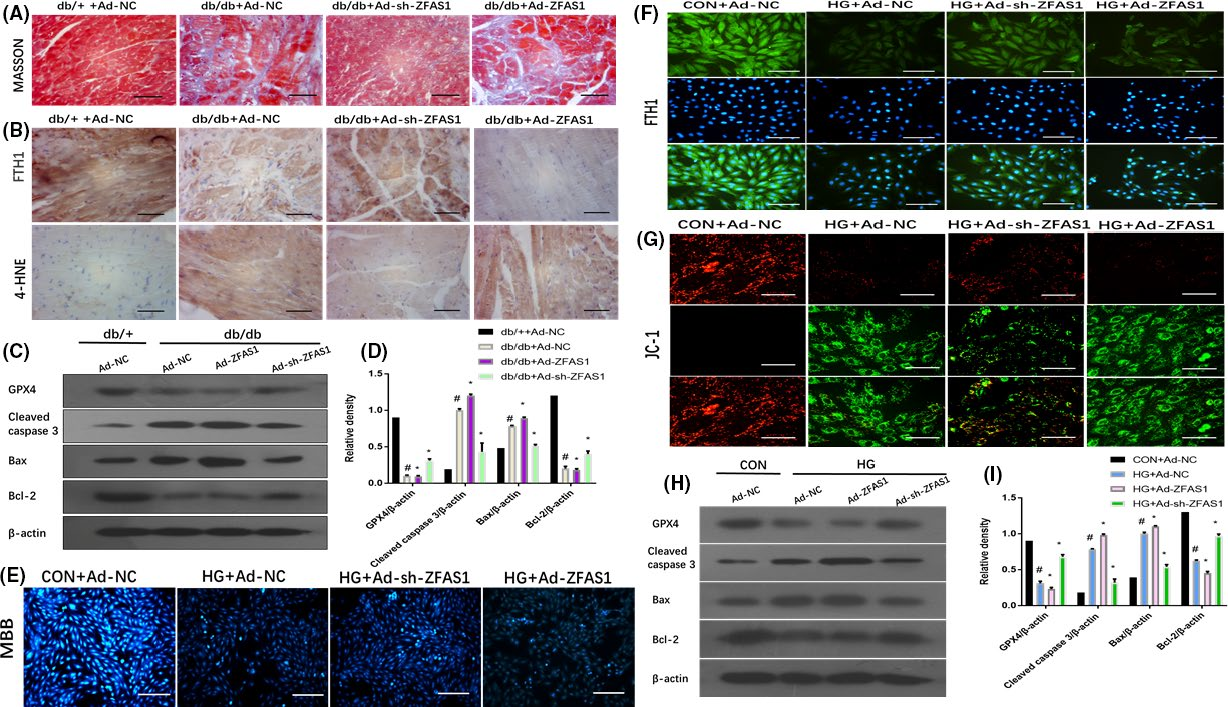
Inhibition of ZFAS1 alleviated ferroptosis in mice with diabetic cardiomyopathy– and HG‐treated cardiomyocytes. Db/+mice (*n* = 8) and db/db mice (*n* = 8) were injected with Ad‐NC, Ad‐ZFAS1 and Ad‐sh‐ZFAS1. Cardiomyocytes were transfected with Ad‐NC, Ad‐ZFAS1 and Ad‐sh‐ZFAS1 with or without HG stimulation. (A) Masson staining was used to assess collagen deposition of the left ventricular myocardial tissues in experimental mice (blue indicates collagen deposition). (B) Relative expression of FTH1 and 4‐HNE was determined by immunohistochemical staining of the left ventricular myocardial tissues of experimental mice. (C‐D) Relative protein expression of GPX4, cleaved caspase‐3, Bax and Bcl‐2 in the left ventricular myocardial tissues of experimental mice was assessed by Western blotting. (E) MBB staining was used to assess GSH levels in experimental cardiomyocytes. (F) Immunofluorescence against FTH1 (green) in experimental cardiomyocytes. (G) JC‐1 staining was used to assess mitochondrial membrane potential in experimental cardiomyocytes. (H–I) Relative protein expression of GPX4, cleaved caspase‐3, Bax and Bcl‐2 in experimental cardiomyocytes was assessed by Western blot analysis. #*p* < 0.05 vs CON group or db/+ group, *p < 0.05 versus HG + Ad‐NC or db/db +Ad‐NC; data are expresses as mean ±standard deviation (SD, *n* = 3)

To further identify the function of ZFAS1 in the DbCM process, we injected Ad‐ZFAS1 and Ad‐sh‐ZFAS1 into the left ventricular free wall of mice. Masson staining shows that collagen deposition was significantly decreased in the left ventricular myocardial tissues of mice in the db/db + Ad‐ZFAS1 group (Figure 3A). Inhibition of ZFAS1 restored the expression of FTH1, reduced the expression of 4‐HNE, as evaluated by immunohistochemical staining (Figure 3B), rescued the expression of GPX4 and inhibited the expression of apoptosis‐related genes, including cleaved caspase 3, Bax and Bcl‐2, as determined by Western blotting (Figure 3C, D). Based on MBB staining, the inhibition of ZFAS1 in HG‐treated cardiomyocytes increased intracellular GSH levels, to a certain extent (Figure 3E), restored the distribution of FTH1 in the cytoplasm (Figure 3F), alleviated the mitochondrial membrane potential, as revealed by the transition from red fluorescence to green fluorescence and measured by JC‐1 staining (Figure 3G), rescued the expression of GPX4 and inhibited the expression of apoptosis‐related genes, including cleaved caspase 3, Bax and Bcl‐2, as measured by Western blotting (Figure 3H, I). These findings suggest that ZFAS1 inhibition prevented ferroptosis in mice with DbCM‐ and HG‐treated cardiomyocytes.

### 3.3 lncRNA‐ZFAS1 can bind with miR‐150‐5p to regulate the expression of CCND2

**FIGURE 4:**
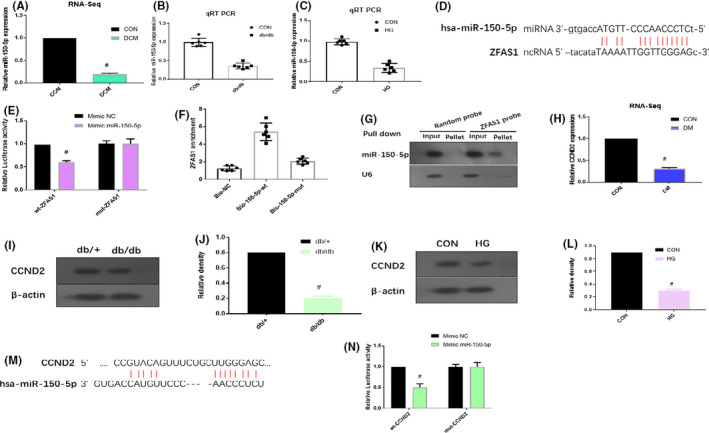
ZFAS1 can bind with miR‐150‐5p to regulate the expression of CCND2. (A) miR‐150‐5p expression levels in the control (CON) group and diabetic cardiomyopathy (DbCM) group based on RNA‐sequencing data (GSE44179). (B) RT‐qPCR analysis of miR‐150‐5p expression in the left ventricular myocardial tissues of db/+ and db/db mice. (C) RT‐qPCR analysis of ZFAS1 expression in control cardiomyocytes and HG‐treated cardiomyocytes. (D) The predicted binding site of miR‐150‐5p with ZFAS1 based on TargetScan program. (E) Dual‐luciferase reporter assay was used to confirm the binding of miR‐150‐5p with ZFAS1. (F) miR‐150‐5p can bind directly to ZFAS1 in vivo as demonstrated in the RNA pull‐down assay. (G) ZFAS1 can bind to miR‐150‐5p in vivo performed by RNA pull‐down assay. (H) CCND2 expression levels in the ventricles of control rats and STZ‐injected rats based on the RNA‐sequencing data (GSE4745). (I, J) Relative protein expression of CCND2 by Western blot analysis in the left ventricular myocardial tissues of db/+ and db/db mice based on Western blotting. (K, L) Relative protein expression of CCND2 in control cardiomyocytes and HG‐treated cardiomyocytes based on Western blot analysis. (M) The predicted binding site of CCND2 with ZFAS1 based on TargetScan program. (N) Dual‐luciferase reporter assay was used to confirm the binding of CCND2 with ZFAS1. #*p* < 0.05 versus CON group or db/+ group; data are expressed as mean ±standard deviation (SD, *n* = 3)

The differential role of the micro‐RNA profiles in the pathophysiology of DbCM was screened using the GSE44179 data set from the GEO database, and miR‐150‐5p was found to be substantially reduced (Figure 4A). To determine whether miR‐150‐5p is involved in DbCM, the expression level of miR‐150‐5p in the left ventricular myocardial tissues of db/db mice and HG‐treated cardiomyocytes was determined by qRT‐PCR. Accordingly, miR‐150‐5p was found to be significantly downregulated in the same manner (Figure 4B, C).

To highlight the potential molecular mechanism by which ZFAS1 and miR‐150‐5p regulate DM, we explored the underlying target binding sites of ZFAS1 and miR‐150‐5p. The predicted binding sites of miR‐150‐5p and ZFAS1 are displayed in Figure 4D. These sites were also analysed using TargetScan software. Based on the dual‐luciferase reporter assay, transfection with miR‐150‐5p mimics significantly reduced the relative firefly luciferase activity of wt‐ZFAS1; however, the mut‐ZFAS1 luciferase activity remained unaffected (Figure 4E). Further, based on a biotin‐avidin pull‐down assay performed to determine whether miR‐150‐5p could directly bind to ZFAS1, ZFAS1 was found to be pulled down by biotinylated wild‐type miR‐150‐5p. Additionally, miR‐150‐5p could not pull down ZFAS1 upon introduction of the miR‐150‐5p mutations that destroy base pairing between ZFAS1 and miR‐150‐5p. These findings indicate that the identification of miR‐150‐5p and ZFAS1 is sequence‐specific (Figure 4F). An inverse pull‐down assay was also performed to determine whether ZFAS1 could pull down miR‐150‐5p, and miR‐150‐5p could thus be co‐precipitated by ZFAS1 using a biotin‐labelled specific ZFAS1 probe (Figure 4G).

Analysis of the GSE44179 data set from the GEO database revealed that CCND2 was significantly decreased in rat ventricles after STZ injection (Figure 4H). To determine whether CCND2 is involved in DbCM, the expression level of CCND2 in the left ventricular myocardial tissues of db/db mice and HG‐treated cardiomyocytes was determined by Western blotting. CCND2 expression was significantly downregulated in a similar manner (Figure 4I–L). TargetScan provided information on the predicted binding sites of miR‐150‐5p and CCND2 (Figure 4M). Based on the dual‐luciferase reporter assay, transfection with the miR‐150‐5p mimics significantly reduced the relative firefly luciferase activity of wt‐CCND2; however, the mut‐CCND2 luciferase activity remained unaffected (Figure 4N). These findings suggest that ZFAS1 can bind with miR‐150‐5p to regulate the expression of CCND2.

### 3.4 ZFAS1 promotes ferroptosis in mice with DbCM and HG‐treated cardiomyocytes by modulating miR‐150‐5p

**FIGURE 5:**
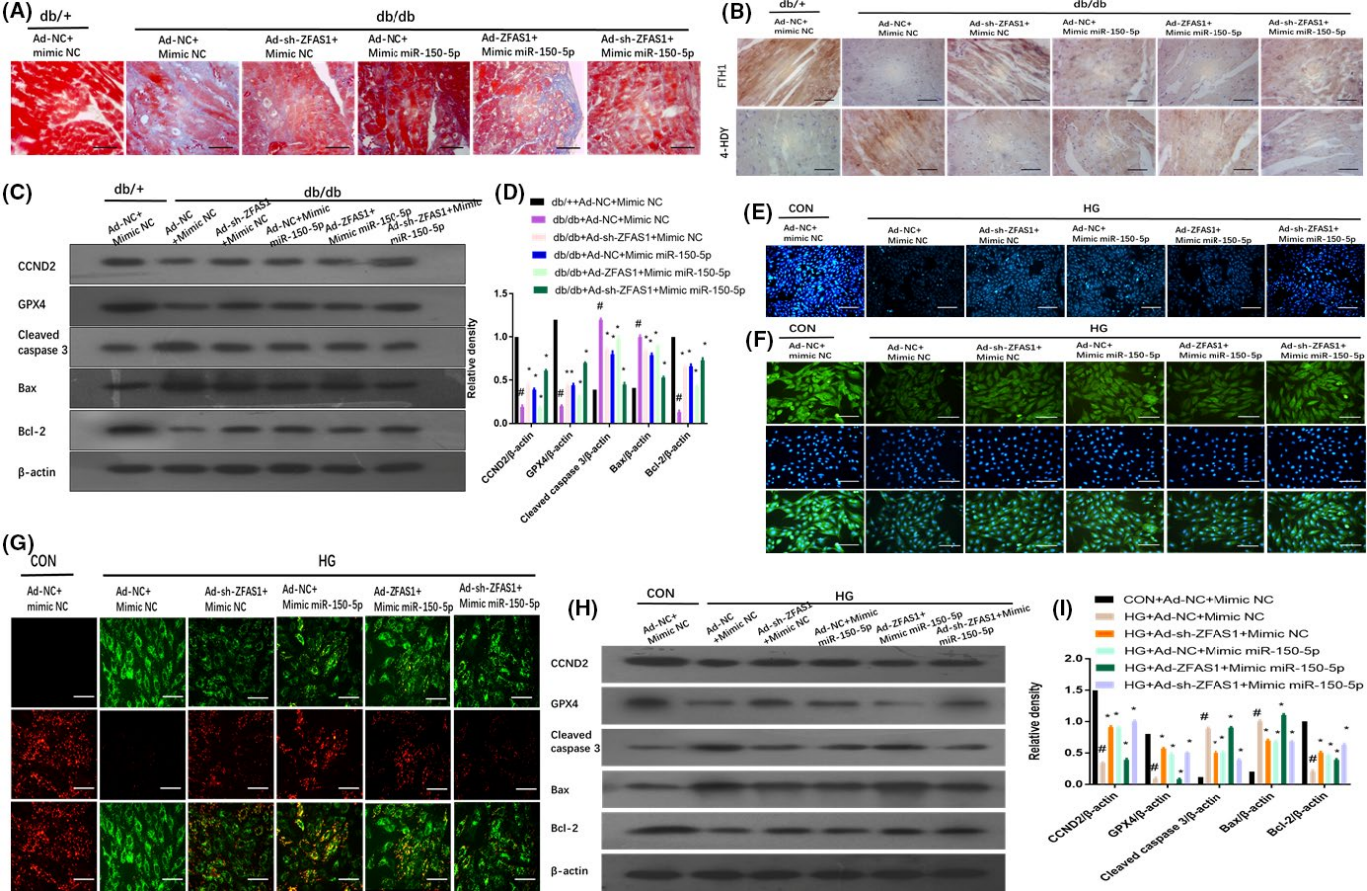
Inhibition of ZFAS1 repressed ferroptosis by up‐regulating miR‐150‐5p in mice with diabetic cardiomyopathy– and HG‐treated cardiomyocytes. Db/+mice (*n* = 8) and db/db mice (*n* = 8) were intracoronarily administered Ad‐NC, Ad‐ZFAS1, Ad‐sh‐ZFAS1, mimic NC, and mimic miR‐150‐5p. Cardiomyocytes were transfected with Ad‐NC, Ad‐ZFAS1, Ad‐sh‐ZFAS1, mimic NC or mimic miR‐150‐5p with or without HG stimulation. (A) Masson staining was used to assess collagen deposition in the left ventricular myocardial tissues of experimental mice (blue indicates collagen deposition). (B) Relative expression of FTH1 and 4‐HNE was determined by immunohistochemical staining of the left ventricular myocardial tissues of experimental mice. (C, D) Relative protein expression of GPX4, cleaved caspase‐3, Bax and Bcl‐2 in the left ventricular myocardial tissues of experimental mice was assessed by Western blotting. (E) MBB staining was used to assess GSH levels in experimental cardiomyocytes. (F) Immunofluorescence against FTH1 (green) in experimental cardiomyocytes. (G) JC‐1 staining was used to assess mitochondrial membrane potential in experimental cardiomyocytes. (H, I) Relative protein expression of GPX4, cleaved caspase‐3, Bax and Bcl‐2 in experimental cardiomyocytes was assessed by Western blot analysis. #*p* < 0.05 versus CON group + Ad‐NC +mimic NC or db/+ + Ad‐NC + mimic NC group, *p < 0.05 versus HG + Ad‐NC + mimic NC or db/db + Ad‐NC + mimic NC; data are expressed as mean ± standard deviation (SD, *n* = 3)

Owing to the interaction between ZFAS1 and miR‐150‐5p, we sought to determine whether ZFAS1 could regulate ferroptosis through miR‐150‐5p. As shown in Figure 5A, miR‐150‐5p stimulation significantly decreased collagen deposition in the left ventricular myocardial tissues of db/db mice, consistent with the function of ZFAS1 inhibition. However, Masson staining showed that stimulation with ZFAS1 abolished the positive effect of miR‐150‐5p. The overexpression of miR‐150‐5p restored the expression of FTH1 and reduced the expression of 4‐HNE (a function of ZFAS1 inhibition), as demonstrated by immunohistochemical staining. However, the overexpression of ZFAS1 could offset the positive effects of miR‐150‐5p (Figure 5B). The overexpression of miR‐150‐5p was found to rescue the expression of GPX4 and CCND2 and inhibit the expression of apoptosis‐related genes, including cleaved caspase‐3, Bax and Bcl‐2, as measured by Western blotting, which is consistent with the function of ZFAS1. However, the overexpression of ZFAS1 counteracted the positive effect of miR‐150‐5p (Figure 5C, D).

Next, we determined whether ZFAS1 regulates ferroptosis through miR‐150‐5p in HG‐treated cardiomyocytes. As shown in Figure 5E, HG‐treated cardiomyocytes transfected with Ad‐sh‐ZFAS1 or miR‐150‐5p mimic displayed increased intracellular GSH levels, as assessed by MBB staining (Figure 5E). However, overexpression of ZFAS1 counteracted the positive effect of miR‐150‐5p. Administering Ad‐ZFAS1 significantly reversed the effect of miR‐150‐5p. This was demonstrated by a reduction in FTH1 expression, as detected by immunofluorescence (Figure 5F), a decrease in mitochondrial membrane potential as detected by JC‐1 staining (Figure 5G), a reduction in GPX4 and CCND2 expression, and the expression of apoptosis‐related genes, including cleaved caspase‐3, Bax and Bcl‐2, as measured by Western blotting (Figure 5H–I).

As shown in Table 1, ZFAS1 inhibition and overexpression of miR‐150‐5p improved the cardiac function in the hearts of db/db mice; however, the overexpression of ZFAS1 counteracted the positive effect of miR‐150‐5p, as shown by a significant decrease in LVEF and LVFS and an evident increase in LVEDD and LVESD.

**TABLE 1:** ZFAS1 improved the cardiac function in the hearts of db/db mice by modulating miR‐150‐5p

Group(n=8)	db/+	db/db				
	Ad‐NC + mimic NC	Ad‐NC + Mimic NC	Ad‐sh‐ZFAS1 + Mimic NC	Ad‐NC + Mimic miR‐150‐5p	Ad‐ZFAS1 + Mimic miR‐150‐5p	Ad‐sh‐ZFAS1 + Mimic miR‐150‐5p
LVEF (%)	79.13±2.13	58.32±2.6	66.03±2.03^*#^	65.36±3.29^*#^	59.23±2.62^*#^	68.63±1.46^*#^
LVFS%	39.1±0.9	21.3±3.5	30.1±3.1^*#^	30.3±2.9^*#^	22.3±3.4^*#^	31.6±2.6^*#^
LVESD (mm)	2.4±0.24	3.6±0.42	3.0±0.23^*#^	3.1±0.12^*#^	3.6±0.21^*#^	2.9±0.43^*#^
LVEDD (mm)	3.7±0.4	4.9±0.6	4.1±0.2^*#^	4.1±0.6^*#^	4.9±0.2^*#^	4.0±0.5^*#^
E/A ratio	2.13±0.59	1.51±0.36	1.71±0.21^*#^	1.72±0.42^*#^	1.50±0.24^*#^	1.82±0.41^*#^

Notes

Values are mean ± SD, *p* < 0.05. The data are presented as means and SD. *p < 0.05 versus db/+ + Ad‐NC + mimic NC; #<0.05 versus db/db + Ad‐NC + mimic NC.

Abbreviations: LVEDD, left ventricular end‐diastolic dimension; LVESD, left ventricular end‐systolic dimension; LVEF, left ventricular ejection fraction; LVFS, left ventricular systolic function.

Collectively, these results indicate that the inhibition of ZFAS1 could suppress ferroptosis in DbCM‐ and HG‐treated cardiomyocytes by targeting miR‐150‐5p. Thus, LncRNA‐ZFAS1 acted as a ceRNA to sponge miR‐150‐5p.

### 3.5 ZFAS1 promotes ferroptosis in mice with DbCM‐ and HG‐treated cardiomyocytes by modulating CCND2

**FIGURE 6:**
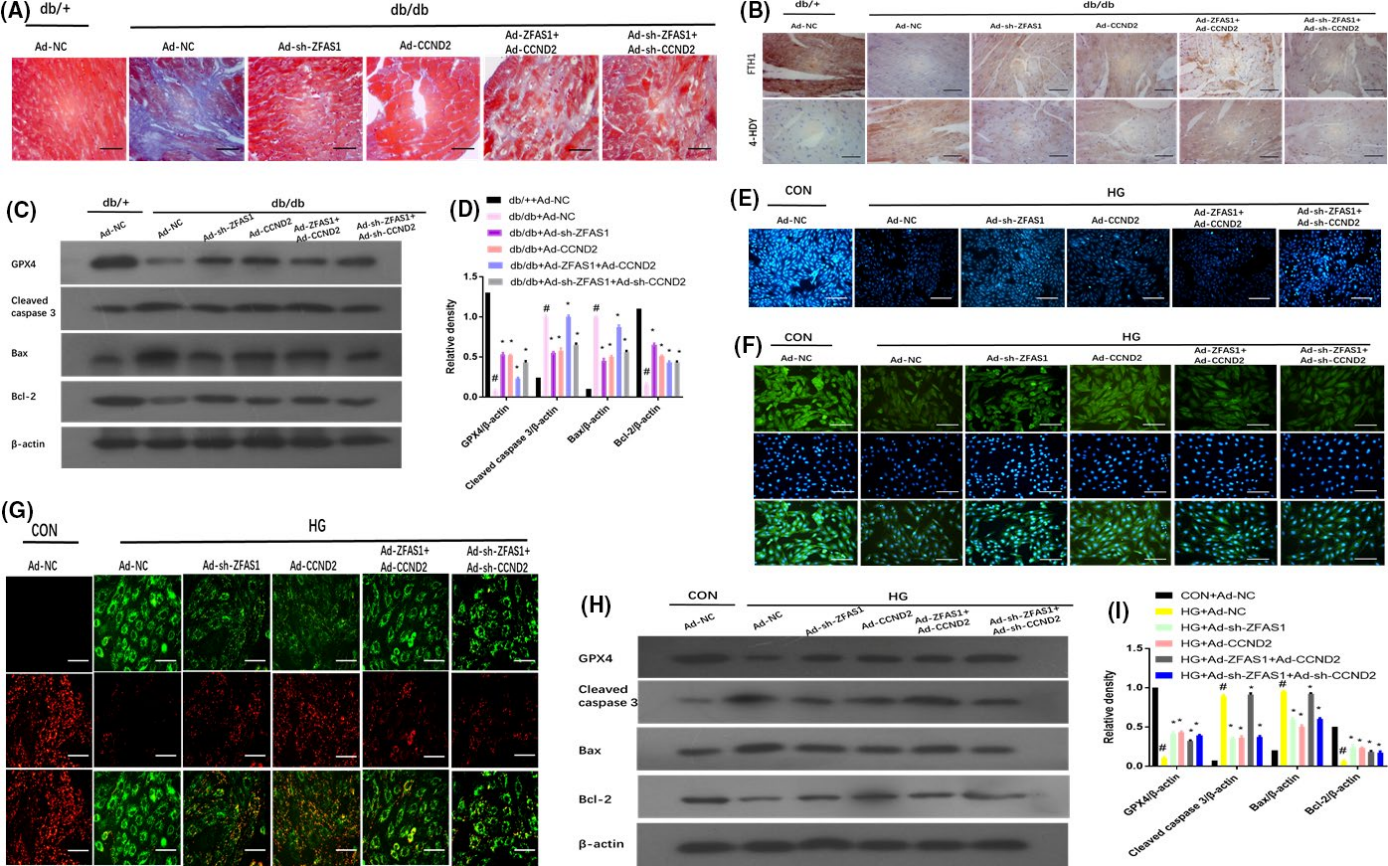
Inhibition of ZFAS1 alleviated ferroptosis by up‐regulating CCND2 in mice with diabetic cardiomyopathy– and HG‐treated cardiomyocytes. Db/+mice (*n* = 8) and db/db mice (*n* = 8) were intracoronarily administered Ad‐NC, Ad‐ZFAS1, Ad‐sh‐ZFAS1, Ad‐CCND2 and Ad‐sh‐CCND2. Cardiomyocytes were transfected with Ad‐NC, Ad‐ZFAS1, Ad‐sh‐ZFAS1, Ad‐CCND2 or Ad‐sh‐CCND2 with or without HG stimulation. (A) Masson staining was used to assess collagen deposition in the left ventricular myocardial tissues of experimental mice (blue indicates collagen deposition). (B) Relative expression of FTH1 and 4‐HNE was determined by immunohistochemical staining of the left ventricular myocardial tissues of experimental mice. (C, D) Relative protein expression of GPX4, cleaved caspase‐3, Bax and Bcl‐2 in the left ventricular myocardial tissues of experimental mice was assessed by Western blotting. (E) MBB staining was used to assess GSH levels in experimental cardiomyocytes. (F) Immunofluorescence against FTH1 (green) in experimental cardiomyocytes. (G) JC‐1 staining was used to assess mitochondrial membrane potential in experimental cardiomyocytes. (H, I) Relative protein expression of GPX4, cleaved caspase‐3, Bax and Bcl‐2 in experimental cardiomyocytes was assessed by Western blot analysis. #*p* < 0.05 versus CON group + Ad‐NC or db/+ + Ad‐NC group, *p < 0.05 versus HG + Ad‐NC or db/db + Ad‐NC; data are expressed as mean ± standard deviation (SD, *n* = 3)

To further examine the role of CCND2 in the positive function of ZFAS1 inhibition against ferroptosis in mice with DbCM‐ and HG‐treated cardiomyocytes, the left ventricular myocardial tissues of db/db mice were administered with Ad‐sh‐ZFAS1, Ad‐ZFAS1, Ad‐CCND2 or Ad‐sh‐CCND2. Interestingly, the overexpression of CCND2 exerted a marked effect on the inhibition of ferroptosis, similar to ZFAS1 inhibition. However, these effects were eliminated when ZFAS1 stimulation was accompanied by an increase in collagen deposition, as determined by Masson staining (Figure 6A), a decrease in FTH1 expression and an increase in 4‐HNE expression, as measured by immunohistochemical staining (Figure 6B), a decrease in GPX4 expression and an increase in the expression of apoptosis‐related genes including cleaved caspase‐3, Bax and Bcl‐2 (Figure 6C, D) in the left ventricular myocardial tissues of db/db mice, as measured by Western blotting. Additionally, the process resulted in a decrease in intracellular GSH levels as assessed by MBB staining (Figure 6E), a reduction in FTH1 expression, as detected by immunofluorescence (Figure 6F), a decrease in mitochondrial membrane potential, as detected by JC‐1 staining (Figure 6G), and a reduction in GPX4 expression and the expression of apoptosis‐related genes, including cleaved caspase‐3, Bax and Bcl‐2, as measured by Western blotting (Figure 6H–I). As shown in Table 2, ZFAS1 inhibition and overexpression of CCND2 could improve cardiac function in the hearts of db/db mice; however, the overexpression of ZFAS1 counteracted the positive effect of CCND2, as shown by a significant decrease in LVEF and LVFS and an evident increase in LVEDD and LVESD. Cumulatively, these findings demonstrated that ZFAS1 inhibition suppressed ferroptosis in mice with DbCM‐ and HG‐treated cardiomyocytes by modulating CCND2. Thus, LncRNA‐ZFAS1 acted as a ceRNA to sponge miR‐150‐5p and downregulate CCND2.

**TABLE 2:** ZFAS1 improved the cardiac function in the hearts of db/db mice by modulating CCND2.

Group (*n* = 8)	db/+	db/db				
	Ad‐NC	Ad‐NC	Ad‐sh‐ZFAS1	Ad‐CCND2	Ad‐ZFAS1+Ad‐CCND2	Ad‐sh‐ZFAS1+Ad‐sh‐CCND2
LVEF (%)	79.18±2.61	58.19±3.2	66.23±2.03^*#^	65.41±2.39^*#^	57.26±3.42^*#^	60.52±1.23^*#^
LVFS%	39.2±0.4	21.4±2.9	30.2±4.1^*#^	30.1±3.2^*#^	21.3±2.8^*#^	21.6±4.5^*#^
LVESD(mm)	2.4±0.31	3.6±0.25	3.0±0.32^*#^	3.1±0.43^*#^	3.7±0.62^*#^	3.7±0.56^*#^
LVEDD(mm)	3.7±0.2	4.9±0.4	4.1±0.1^*#^	4.1±0.4^*#^	4.9±0.3^*#^	4.9±0.2^*#^
E/A ratio	2.14±0.39	1.51±0.35	1.71±0.35^*#^	1.71±0.44^*#^	1.50±0.56^*#^	1.50±0.41^*#^

Notes

Values are mean ± SD, *p* < 0.05. The data are presented as means and SD. *p < 0.05 versus db/+ + Ad‐NC; #<0.05 versus db/db +Ad‐NC.

Abbreviations: LVEDD, left ventricular end‐diastolic dimension; LVESD, left ventricular end‐systolic dimension; LVEF, left ventricular ejection fraction; LVFS, left ventricular systolic function.

## 4 DISCUSSION

Diabetes mellitus is an established independent risk factor for HF; however, HF can accelerate cardiovascular complications associated with diabetes mellitus.^5^ According to a report, diabetes is identified in 15%–35% of patients with HF.^35^ DbCM is also considered to be an indispensable pathophysiological state among patients with diabetes, which could result in dysfunctional cardiomyocytes, abnormal myocardial function and the final cardiac dysfunction characterized by left ventricular longitudinal dysfunction.^8^, ^36^, ^37^ However, establishing a clinical therapy or specific treatment for DbCM is challenging. To date, a molecular mechanism to guide the treatment of DbCM has not been identified. In the present study, we aimed to elucidate the role of lncRNA‐ZFAS1, miR‐150‐5p and CCND2 in the ferroptosis of cardiomyocytes in DbCM. Specifically, inhibition of ZFAS1 could potentially alleviate myocardial fibrosis in DbCM by inhibiting cardiomyocyte ferroptosis by sponging miR‐150‐5p to activate CCND2.

Over the past decade, the roles of lncRNAs have been found to be mainly associated with various cardiovascular diseases, especially MI.^20^, ^38^, ^39^, ^40^ However, little is known about the role of lncRNAs in the pathogenesis of DbCM. The GSE26887 data set from the GEO database revealed that lncRNA‐ZFAS1 was significantly upregulated in diabetic patients with HF compared to that in patients with diabetes (Figure 2A). As expected, ZFAS1 was upregulated in mice with DbCM‐ and HG‐treated cardiomyocytes (Figure 2B, C). ZFAS1 is a cardiac‐related lncRNA. Knockdown of lncRNA‐ZFAS1 has been shown to protect cardiomyocytes from MI.^14^ However, reports have emphasized that ZFAS1 is upregulated to promote cardiac disease^41^ and induce mitochondria‐mediated cardiomyocyte apoptosis.^16^

Ferroptosis, a unique cell death characterized by the stimulation of reactive oxygen species, results in mitochondrial dysfunction, which is induced by iron catalytic activity and lipid peroxidation.^42^ The accumulated evidence demonstrates that ferroptosis is a critical form of cardiomyocyte death.^43^, ^44^ Based on immunohistochemical staining (Figure 2D) and immunofluorescence staining (Figure 2G), the expression levels of the iron storage protein FTH1 were upregulated in the Fer‐1 + db/db and Fer‐1 + HG groups, compared with the db/db and HG groups. Additionally, immunohistochemical staining also revealed the expression of 4‐HNE (Figure 2D). Finally, based on Western blotting, GPX4 was found to be upregulated in the Fer‐1 + db/db and Fer‐1 + HG groups, compared with the db/db and HG groups. These findings suggested that ferroptosis could accumulate in the left ventricular myocardial tissues of mice with DbCM and HG‐treated cardiomyocytes (Figure 2E, F, H, I).

Based on the upregulated expression of FTH1 observed via immunohistochemical staining (Figure 3B) and immunofluorescence staining (Figure 3F); the upregulation of the ferroptosis termination protein, GPX4, revealed by Western blot analysis (Figure 3C, D, 3H, I); the increased intracellular GSH levels indicated via MBB staining (Figure 3E); the restoration of mitochondrial membrane potential demonstrated by JC‐1 staining; all changes related to ferroptosis that led to the reduction of collagen deposition to attenuate cardiac fibrosis in myocardial tissue as determined by Masson staining (Figure 3A); and the decreased expression of apoptosis‐related genes including cleaved caspase 3, Bax and Bcl‐2, as measured by western blotting, inhibition of ZFAS1 in db/db mice could attenuate ferroptosis in the left ventricular myocardial tissues of mice with DbCM‐ and HG‐treated cardiomyocytes (Figure 3C, D, H, I).

Based on emerging evidence, miR‐150‐5p is involved in heart disease and HF.^45^, ^46^ Analysis of the GSE44179 data set from the GEO database and RT‐qPCR of the left ventricular myocardial tissues retrieved from db/db mice and HG‐treated cardiomyocytes revealed that miR‐150‐5p was significantly downregulated (Figure 4A–C). Type D cyclins regulate the transition from the G1 phase to the S phase of the cell cycle; however, the overexpression of CCND2 can activate the cell cycle of cardiomyocytes.^25^, ^47^ Analysis of the GSE4745 data set from the GEO database and RT‐qPCR of the left ventricular myocardial tissues retrieved from db/db mice and HG‐treated cardiomyocytes revealed that CCND2 was significantly downregulated (Figure 4H–L). Furthermore, findings of the RNA pull‐down assay (Figure 4F–G), dual‐luciferase reporter assay (Figure 4E, N) and TargetScan program (Figure 4D, M) suggest that ZFAS1 can bind with miR‐150‐5p to regulate the expression of CCND2.

**FIGURE 7:**
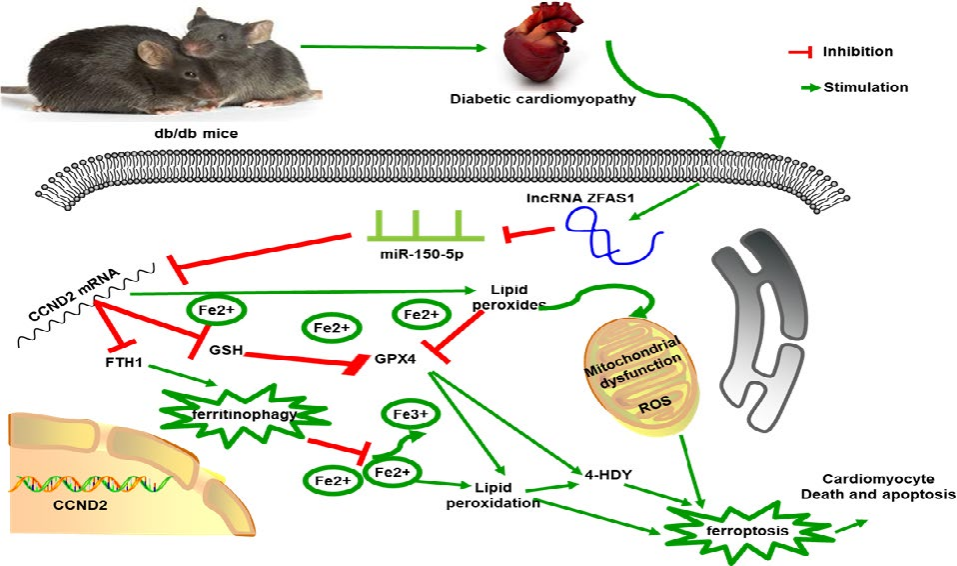
Schematic representing that ZFAS1 was highly expressed in DbCM, and ZFAS1 acts as ceRNA to sponge miR‐150‐5p and could downregulate CCND2 expression, thereby indeed promoting ferroptosis to accelerate DbCM

Herein, the overexpression of miR‐150‐5p and CCND2 significantly alleviated ferroptosis, consistent with the effect of ZFAS1 inhibition observed in db/db mice and HG‐treated cardiomyocytes based on the improvement of cardiac function by a significant increase in LVEF and LVFS and an evident decrease in LVEDD and LVESD (Tables 1, 2) and the downregulated expression of FTH1 observed via immunohistochemical staining (Figures 5B, 6B) and immunofluorescence staining (Figures 5F, 6F), these positive effects were eliminated following ZFAS1 stimulation. Further, Western blotting revealed that GPX4 was downregulated (Figure 5C, D, H, I, 6C, D, H, I), whereas MBB staining revealed a decrease in intracellular GSH levels (Figures 5E, 6E). Based on JC‐1 staining, the mitochondrial membrane potential was reduced (Figures 5G, 6G). According to Masson staining, all changes related to ferroptosis resulted in the accumulation of collagen deposition, which induced cardiac fibrosis in the myocardial tissue (Figures 5A, 6A). Further, the expression of apoptosis‐related genes including cleaved caspase 3, Bax and Bcl‐2 was found to be increased as determined by Western blotting (Figures 5C, D, H, I, 6C, D, H, I). These findings suggest that ZFAS1 inhibition impedes cardiomyocyte ferroptosis by sponging miR‐150‐5p to activate CCND2 against DbCM (Figure 7).

Collectively, all these findings strongly suggest that ZFAS1 simulation promotes ferroptosis in DbCM. Further, lncRNA‐ZFAS1 was recognized to act as a ceRNA to sponge miR‐150‐5p and could downregulate CCND2. Most importantly, ZFAS1 inhibition suppressed cardiomyocyte ferroptosis and attenuated DbCM progression. Targeting lncRNA‐ZFAS1 will enable further development of novel treatments for DbCM.

## CONFLICT OF INTEREST

The authors declare that they have no competing interests.

## AUTHOR CONTRIBUTIONS

**Tingjuan Ni:** Conceptualization (equal); Data curation (equal); Investigation (equal); Resources (equal); Supervision (equal); Writing‐original draft (equal); Writing‐review & editing (equal). **Xingxiao Huang:** Formal analysis (equal); Investigation (equal); Methodology (equal); Software (equal); Supervision (equal). **Sunlei Pan:** Investigation (equal); Project administration (equal); Software (equal). **zhongqiu lu:** Conceptualization (equal); Funding acquisition (equal); Methodology (equal); Supervision (equal); Validation (equal); Visualization (equal); Writing‐review & editing (equal).

## Supporting information

Supplementary Material

Supplementary Material

Supplementary Material

Supplementary Material

Supplementary Material
