# Supplementary material for: Inhibition of the long non‐coding RNA ZFAS1 attenuates ferroptosis by sponging miR‐150‐5p and activates CCND2 against diabetic cardiomyopathy
Source: J Cell Mol Med. 2021 Oct 5;25(21):9995–10007. doi: 10.1111/jcmm.16890 (PMC8572773; doi:10.1111/jcmm.16890)
Supplement: Supplementary file 5 — Supplementary Material [file JCMM-25-9995-s003.docx]

**Figure 2**

**D**

**db/+**

**db/db**

**db/db+Fer-1**


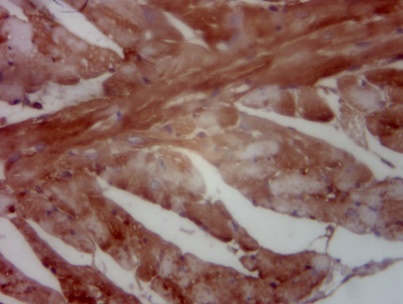

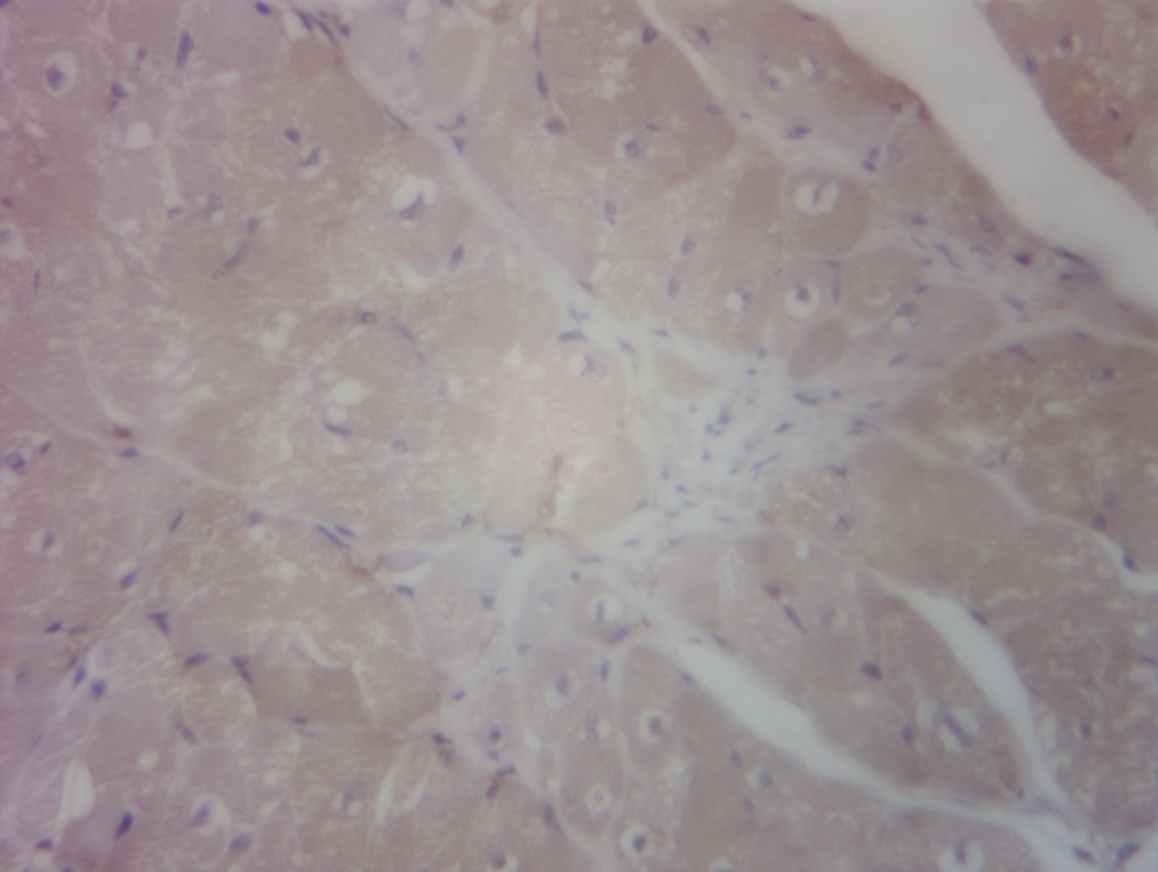

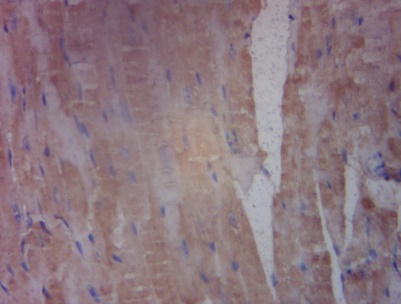


**FTH1**


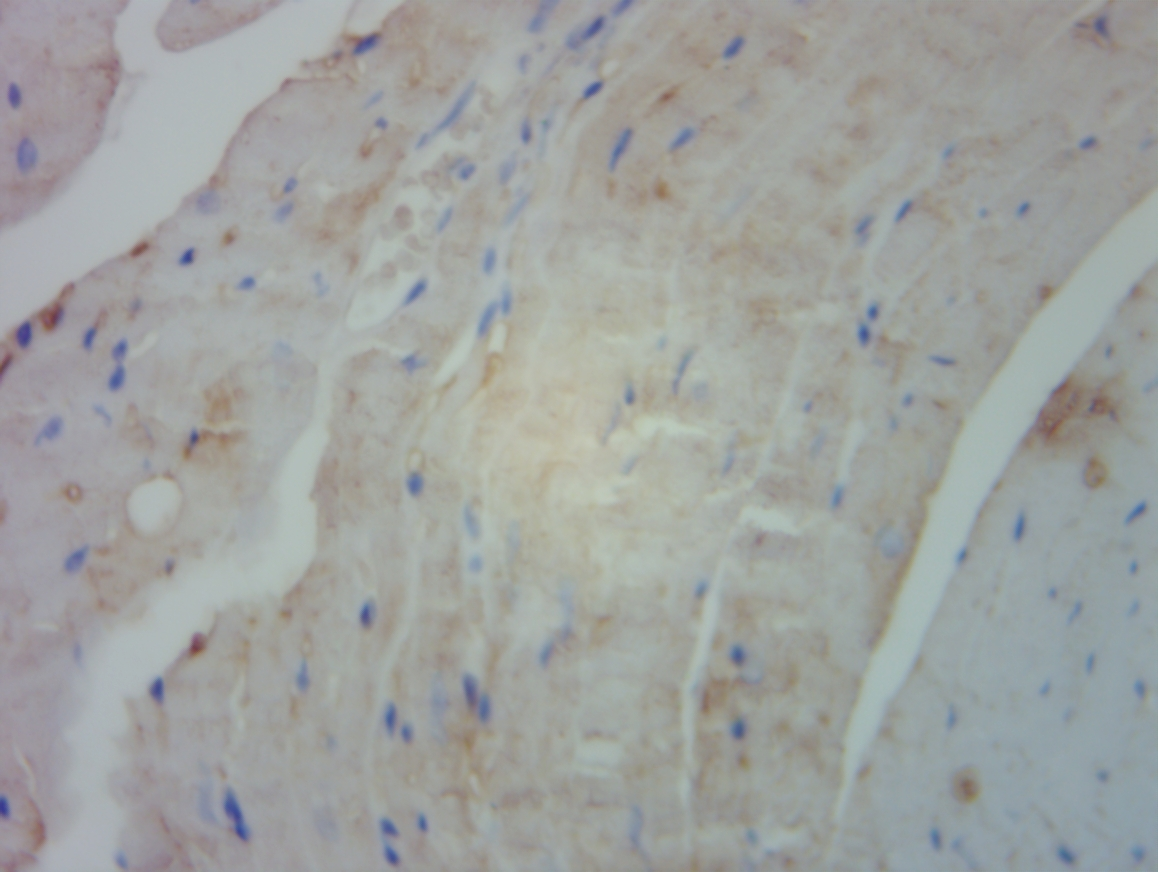

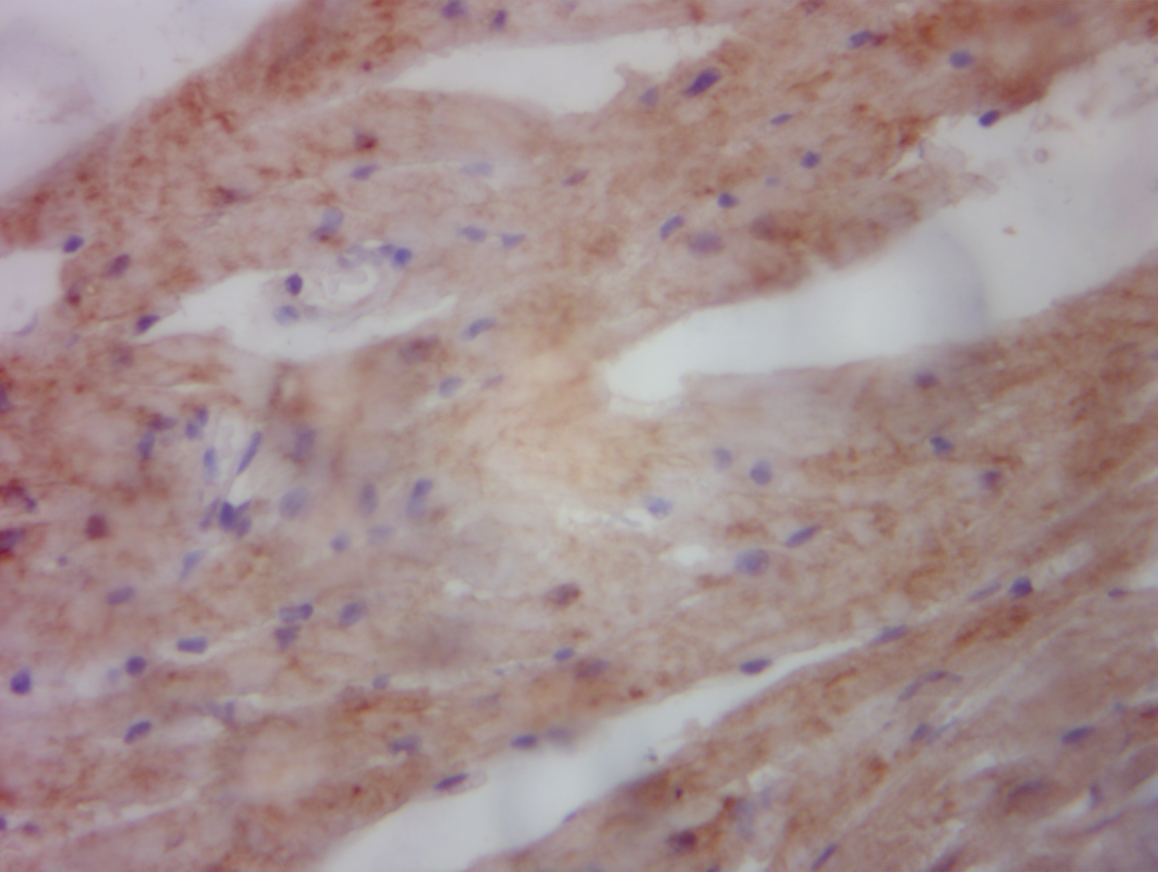

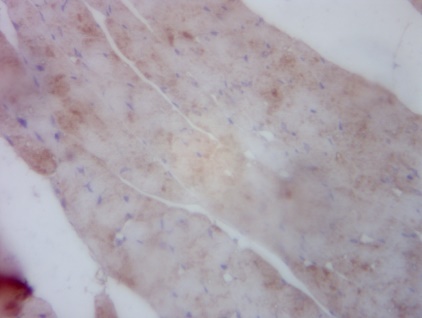


**4-HNE**

**E**

**db/db+Fer-1**

**db/db**

**db/+**


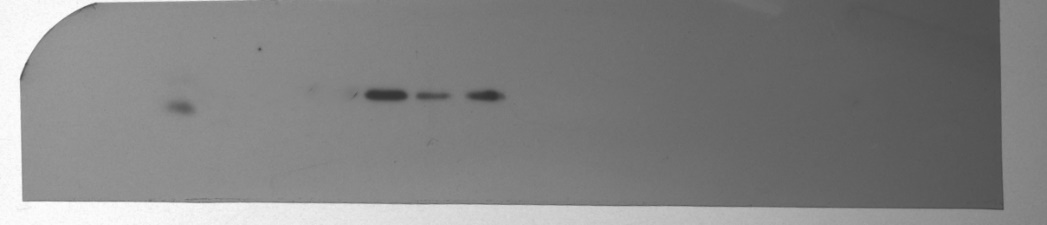


**GPX4**

**β-actin**


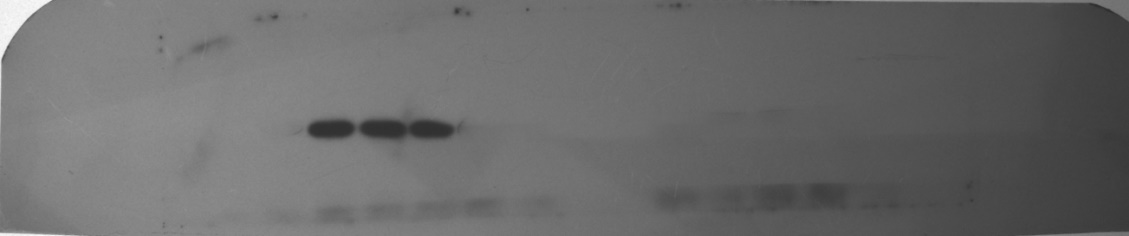


**G**

**FTH1**


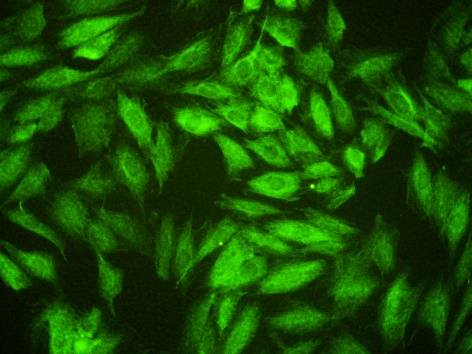

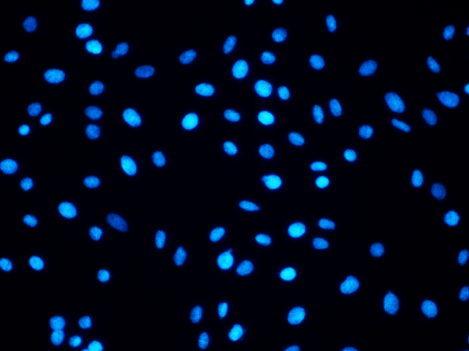


**CON**


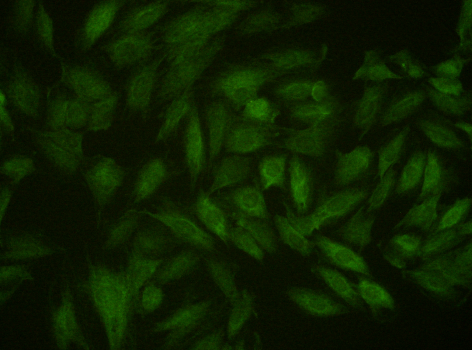

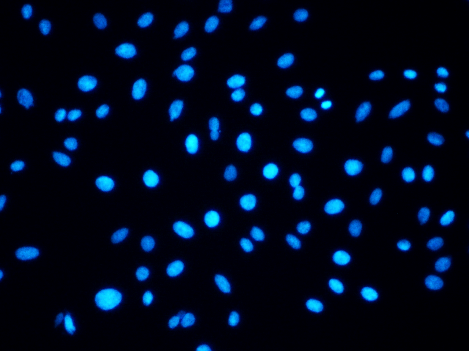


**HG**

**HG+**

**Fer-1**


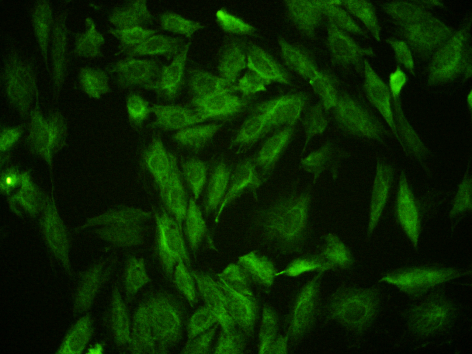

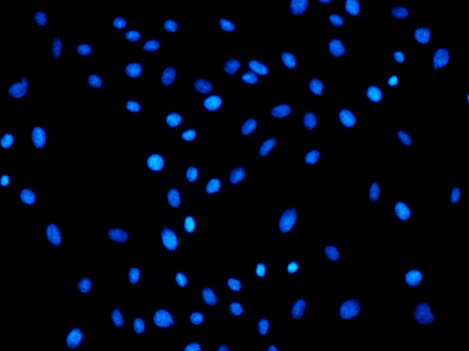


**H**

**CON**

**HG**

**HG+Fer-1**


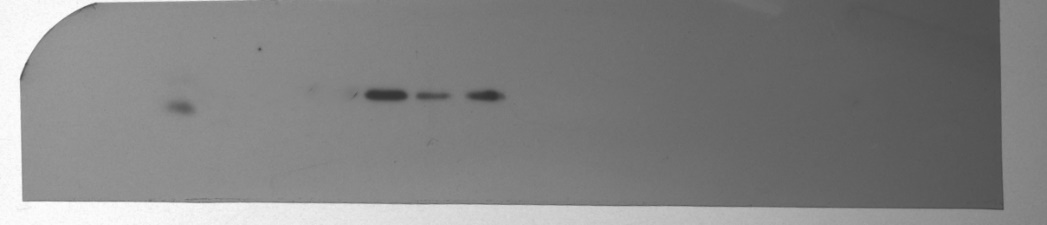


**GPX4**

**β-actin**


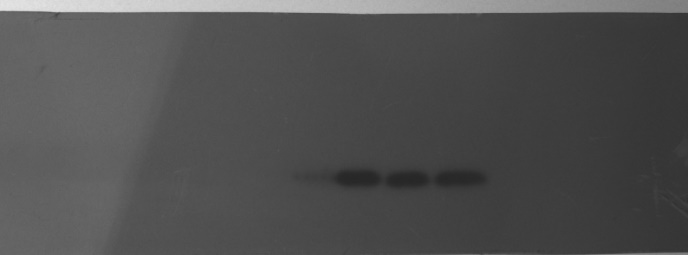


**Figure 3**

**A**

**db/+ +Ad-NC**

**db/db+Ad-NC**


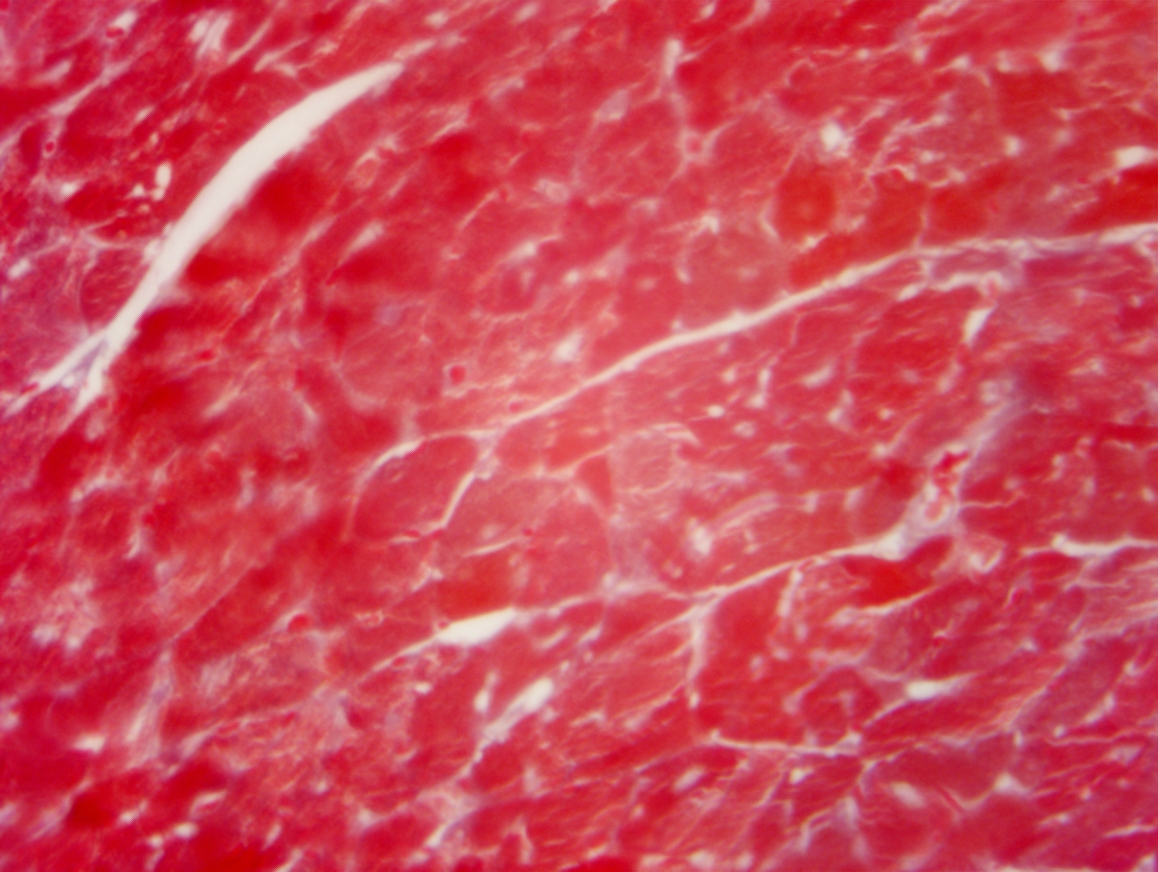

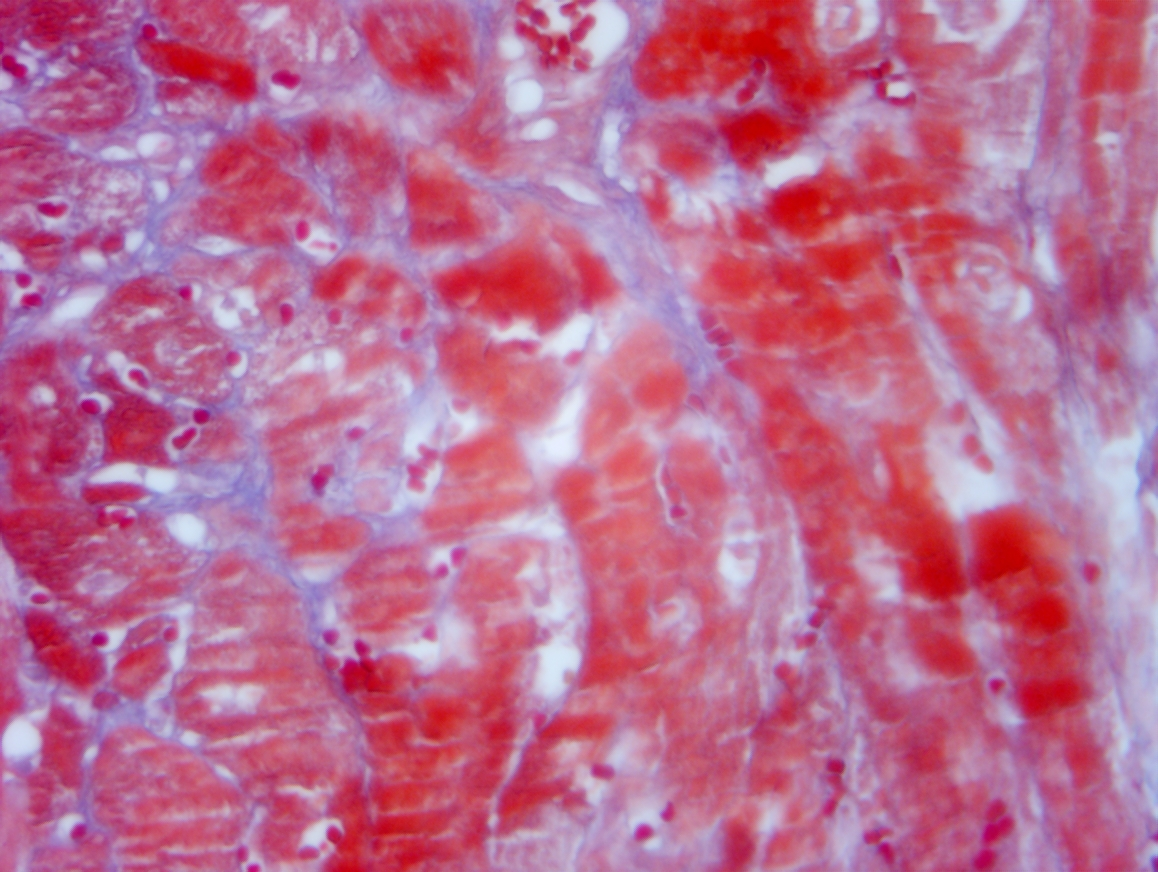


**MASSON**

**db/db+Ad-ZFAS1**

**db/db+Ad-sh-ZFAS1**


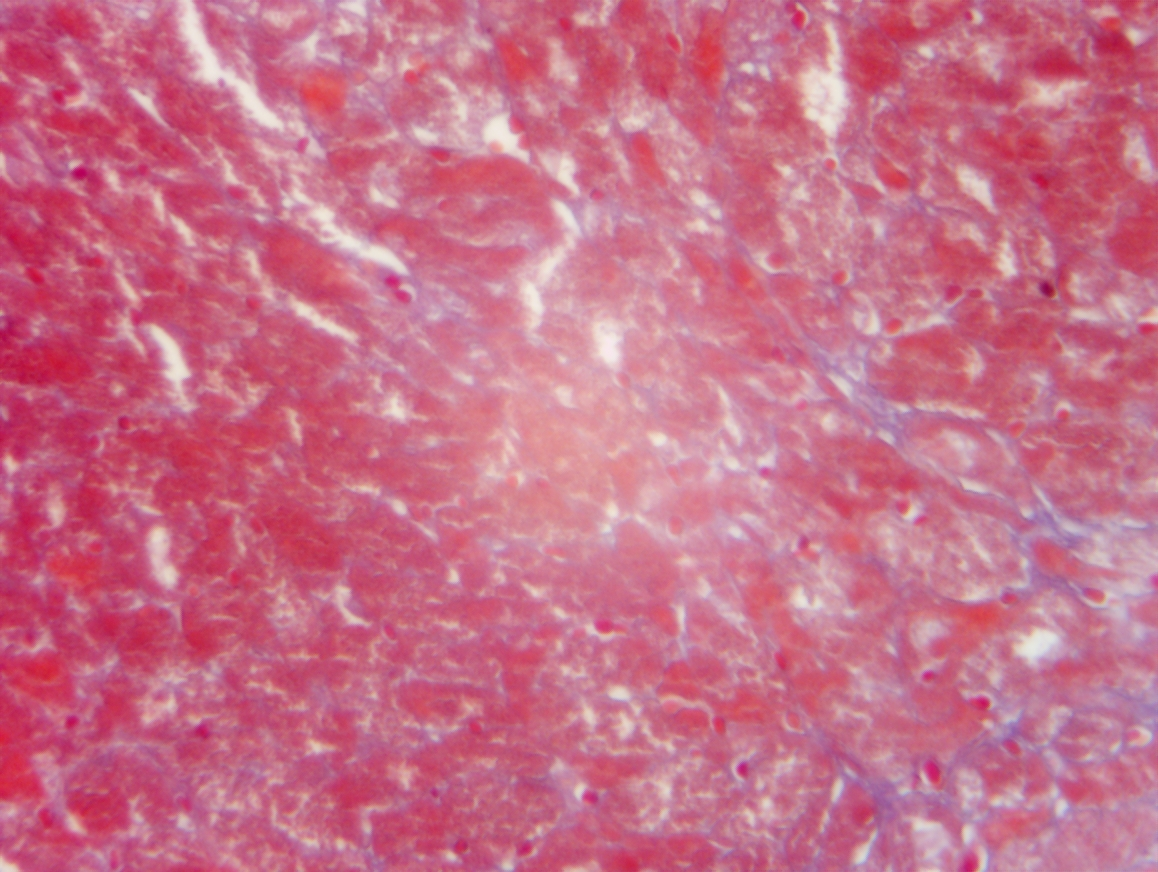

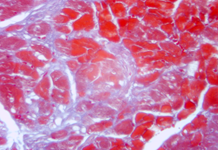


**B**

**db/db+Ad-NC**

**db/+ +Ad-NC**

**FTH1**


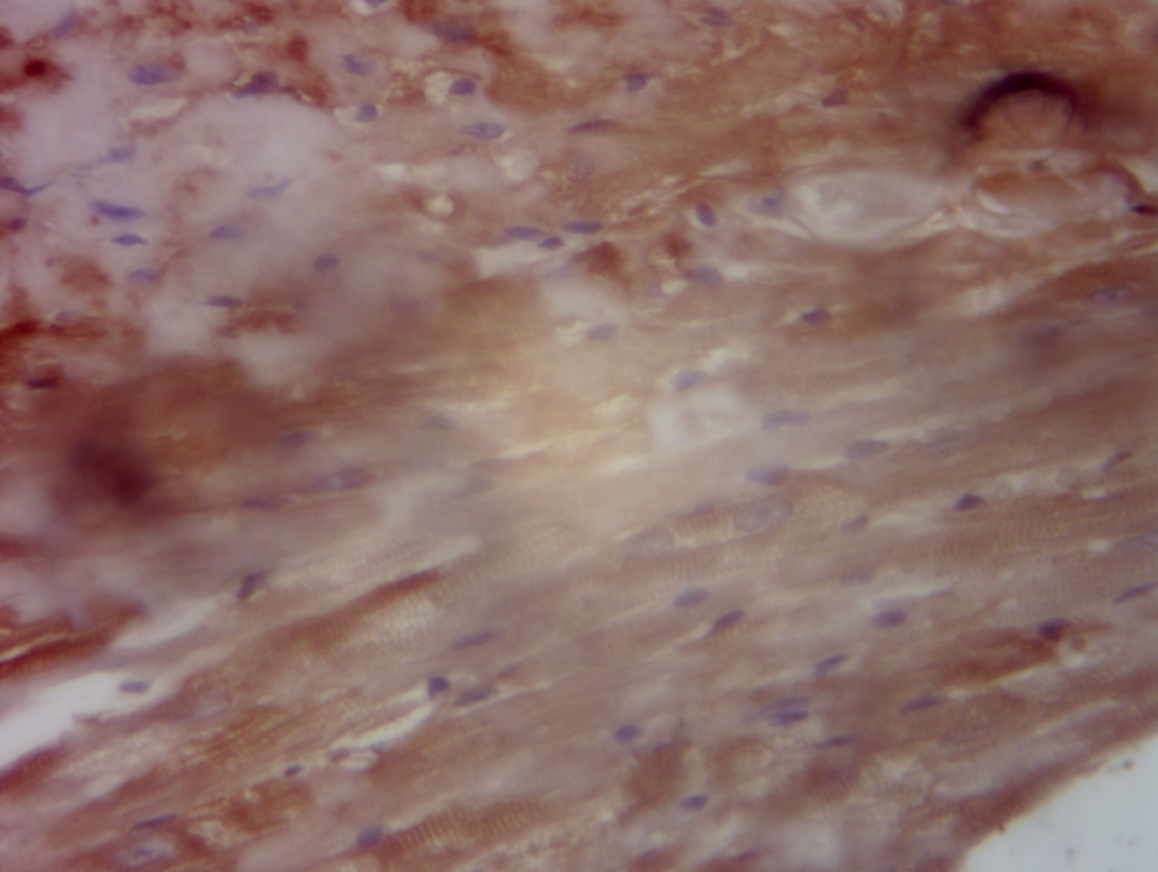

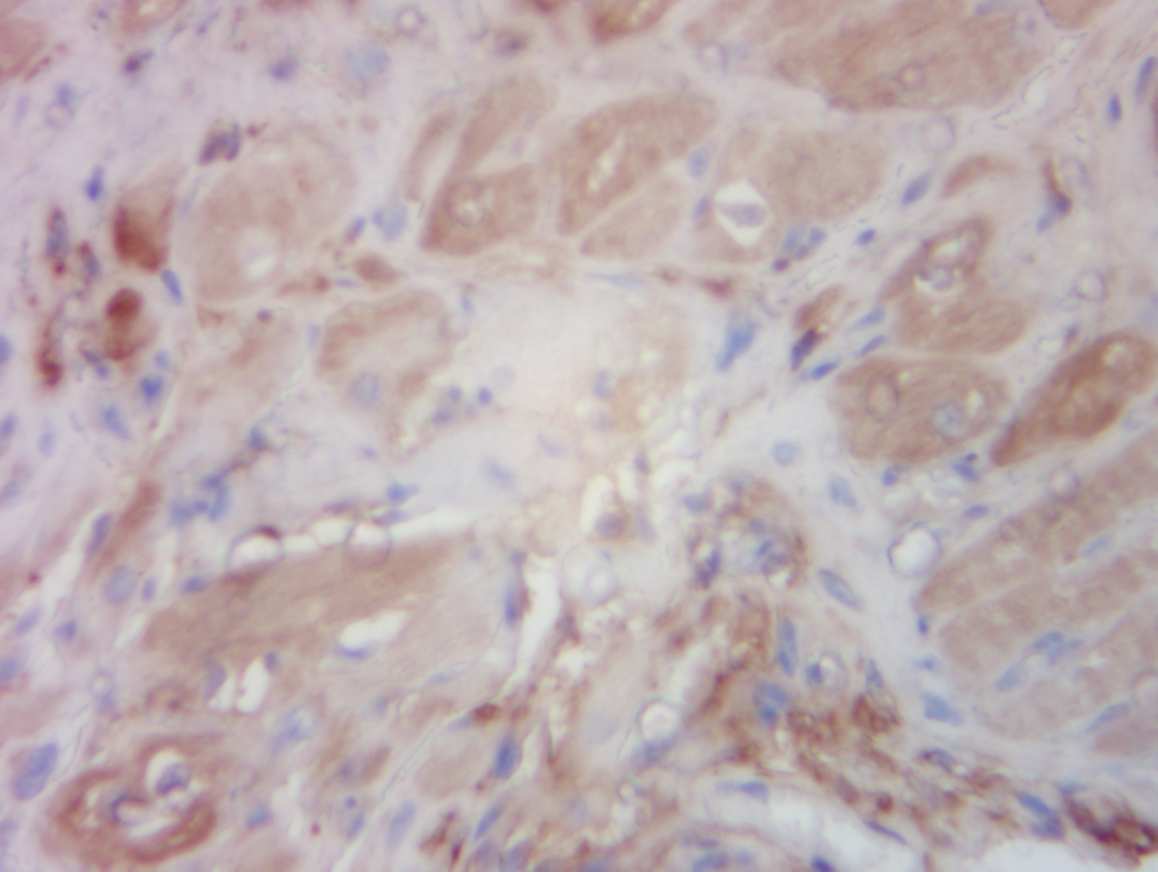


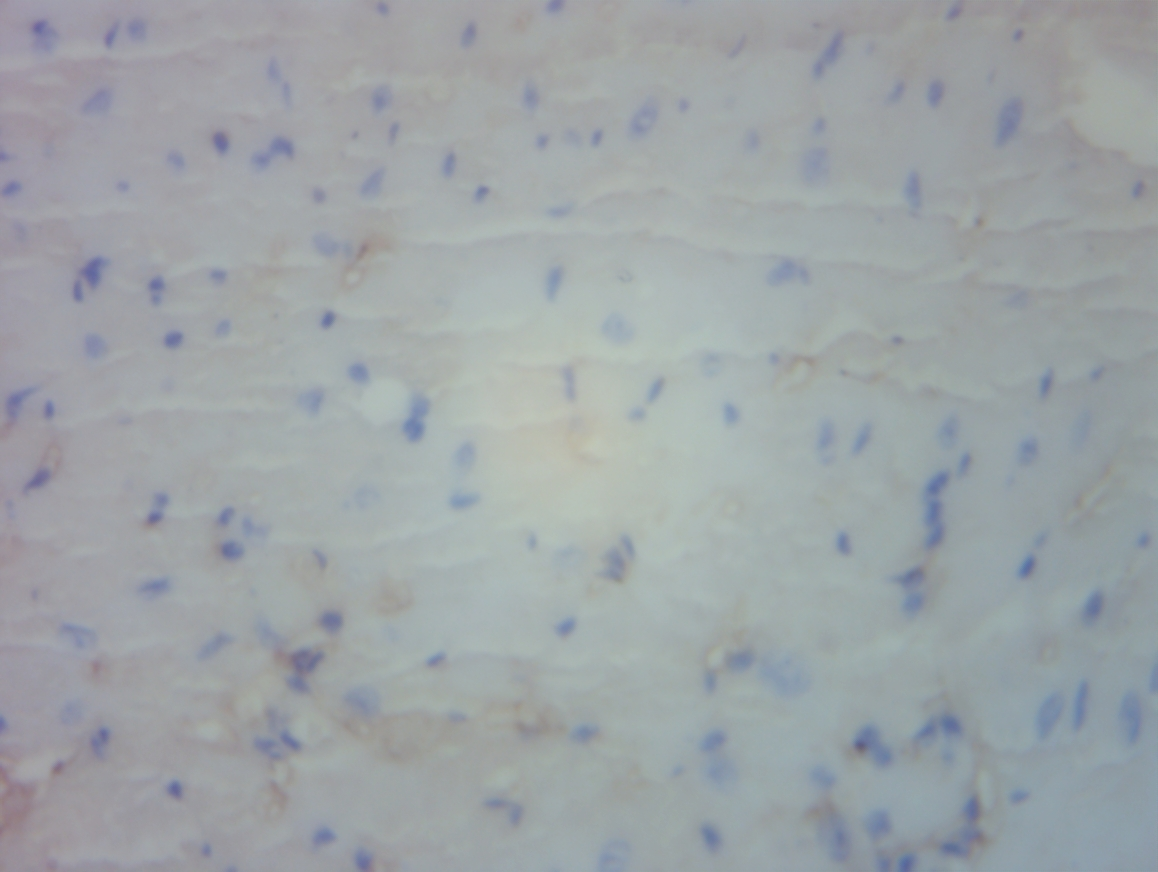

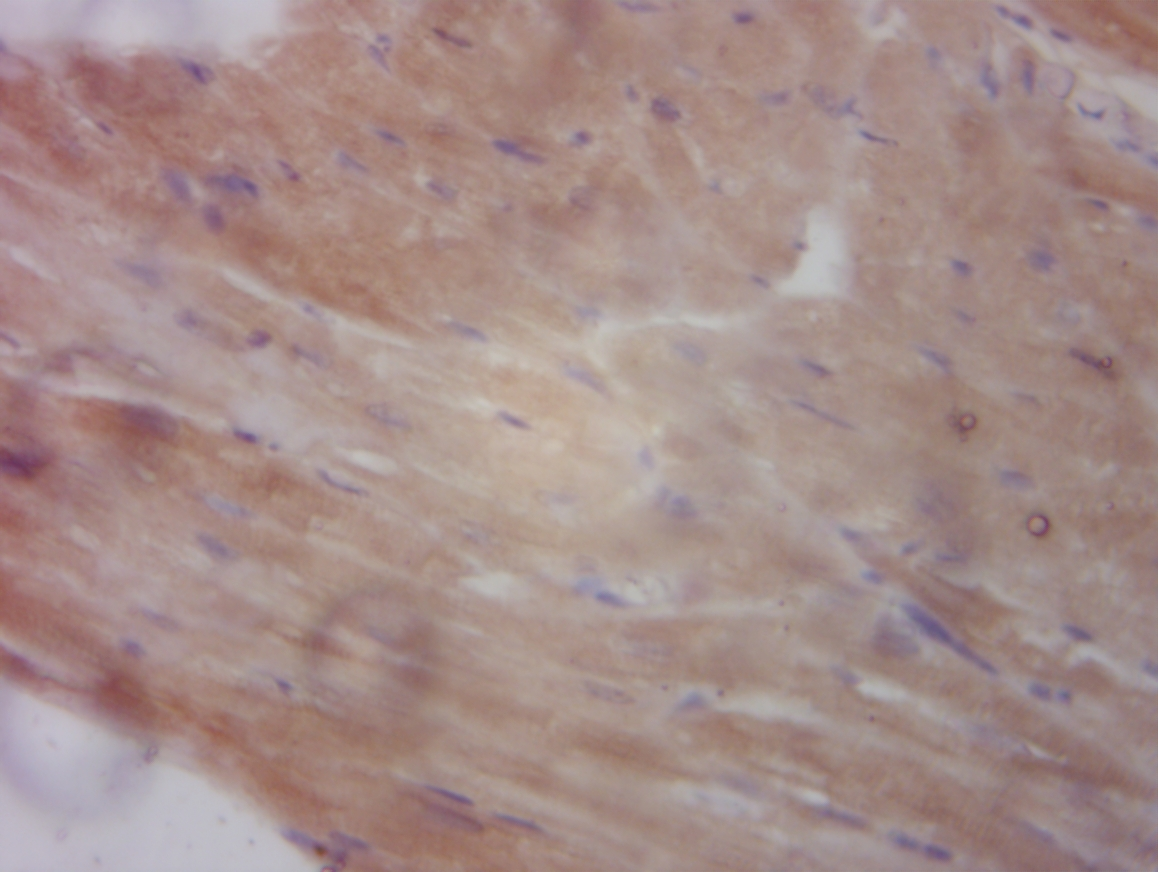


**4-HNE**

**db/db+Ad-ZFAS1**

**db/db+Ad-sh-ZFAS1**


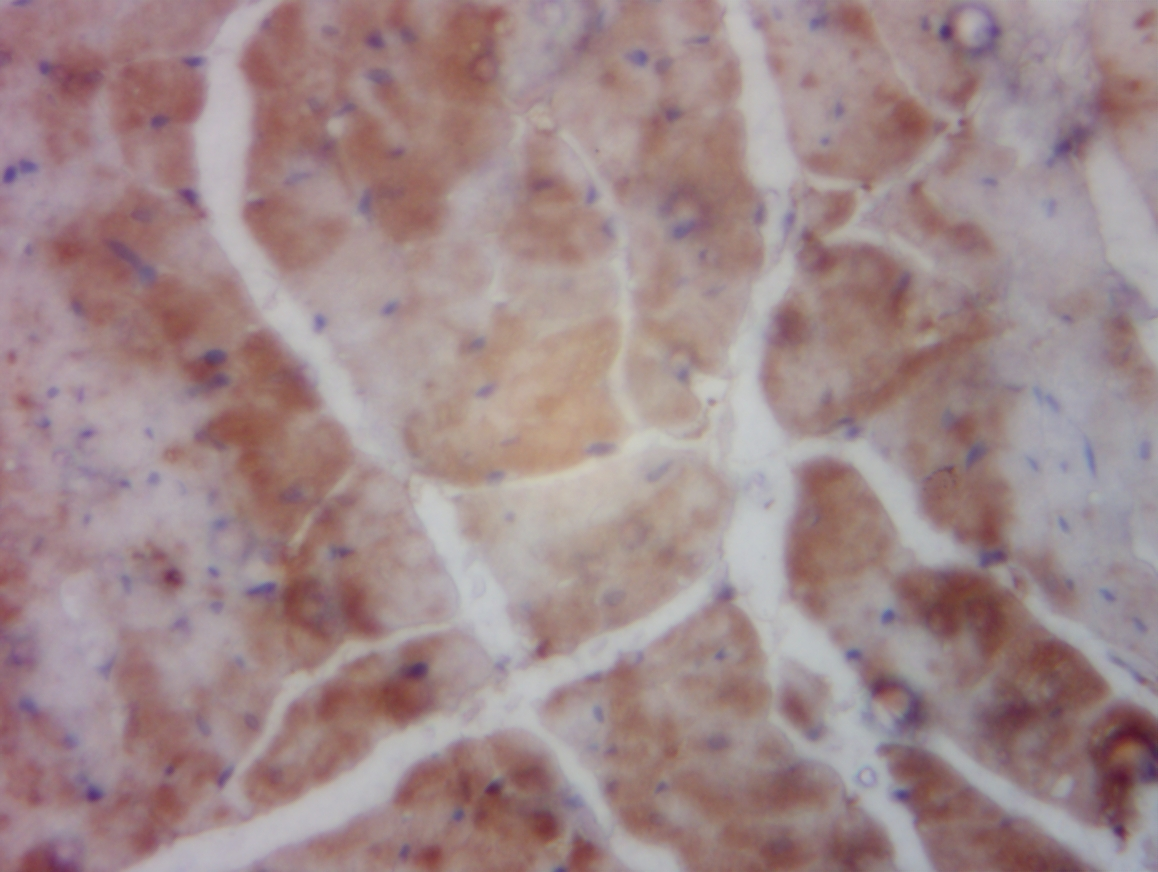

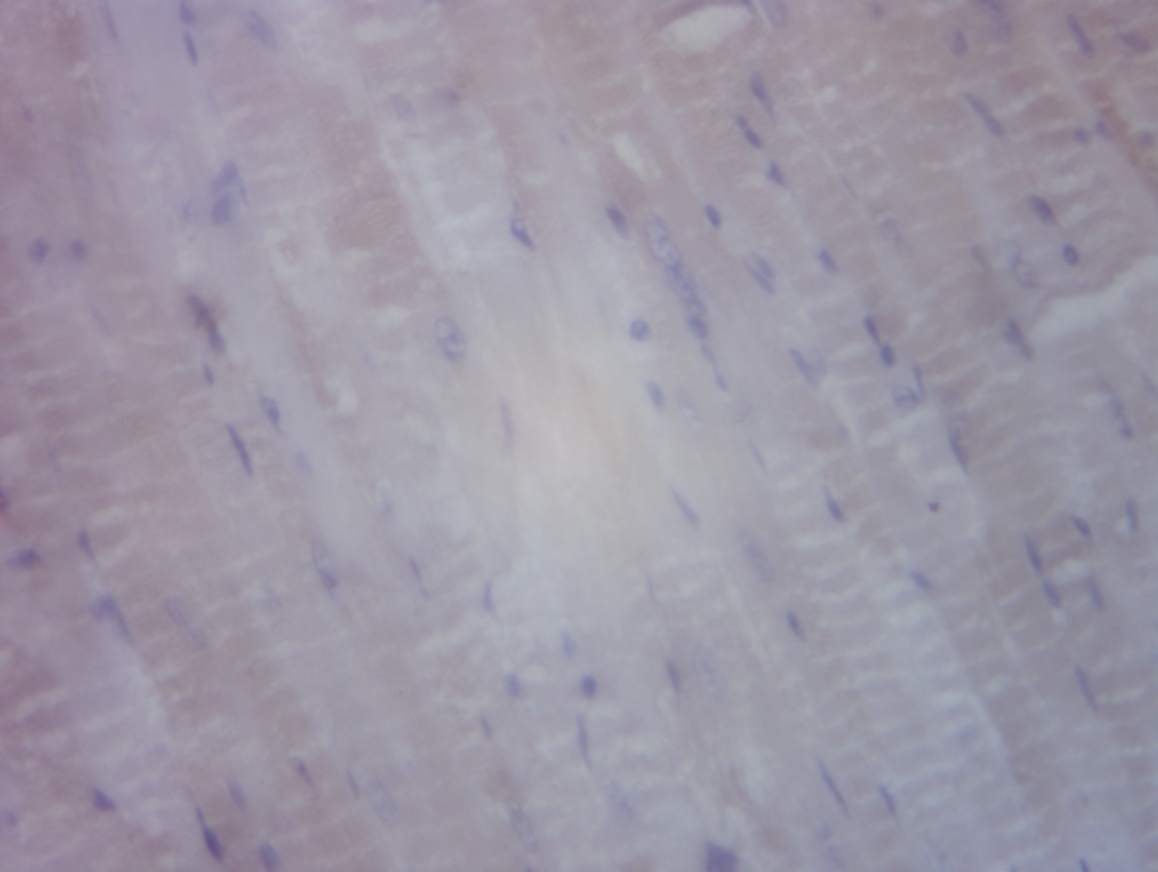


**FTH1**


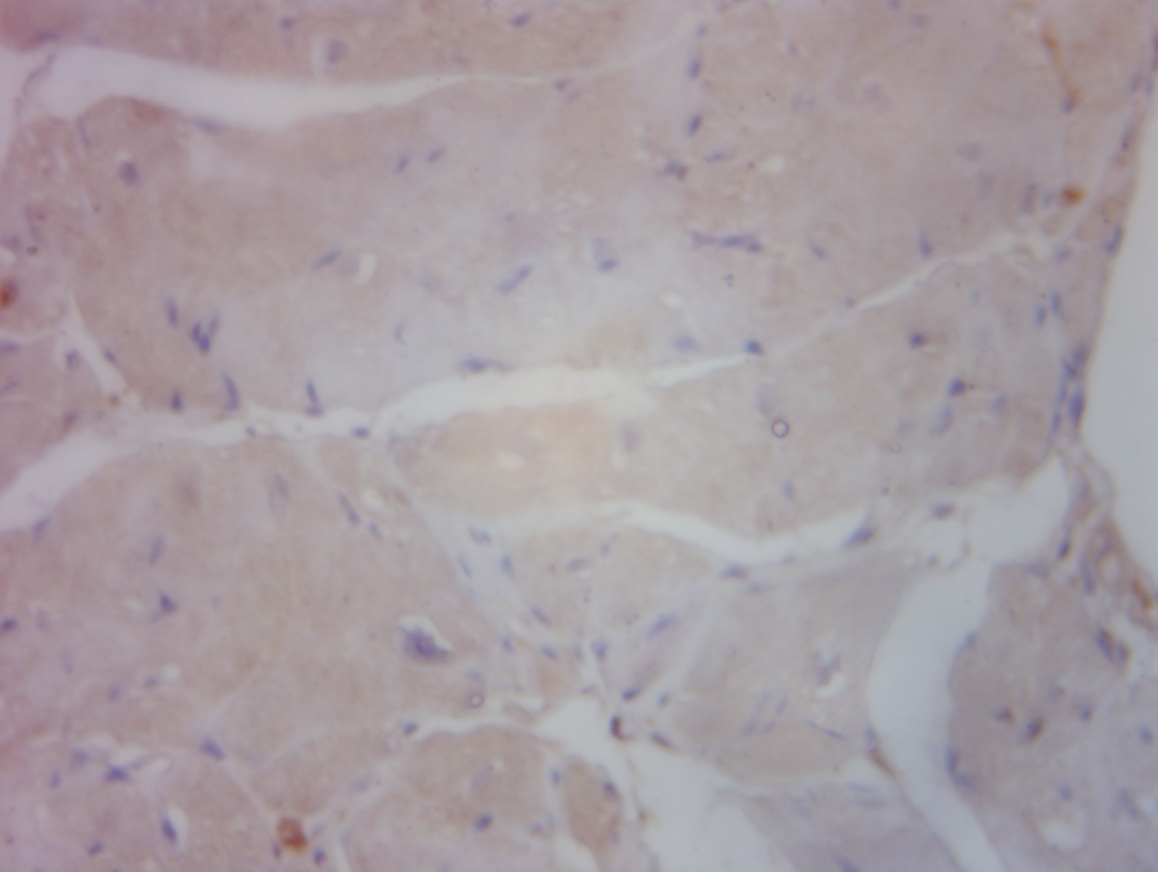

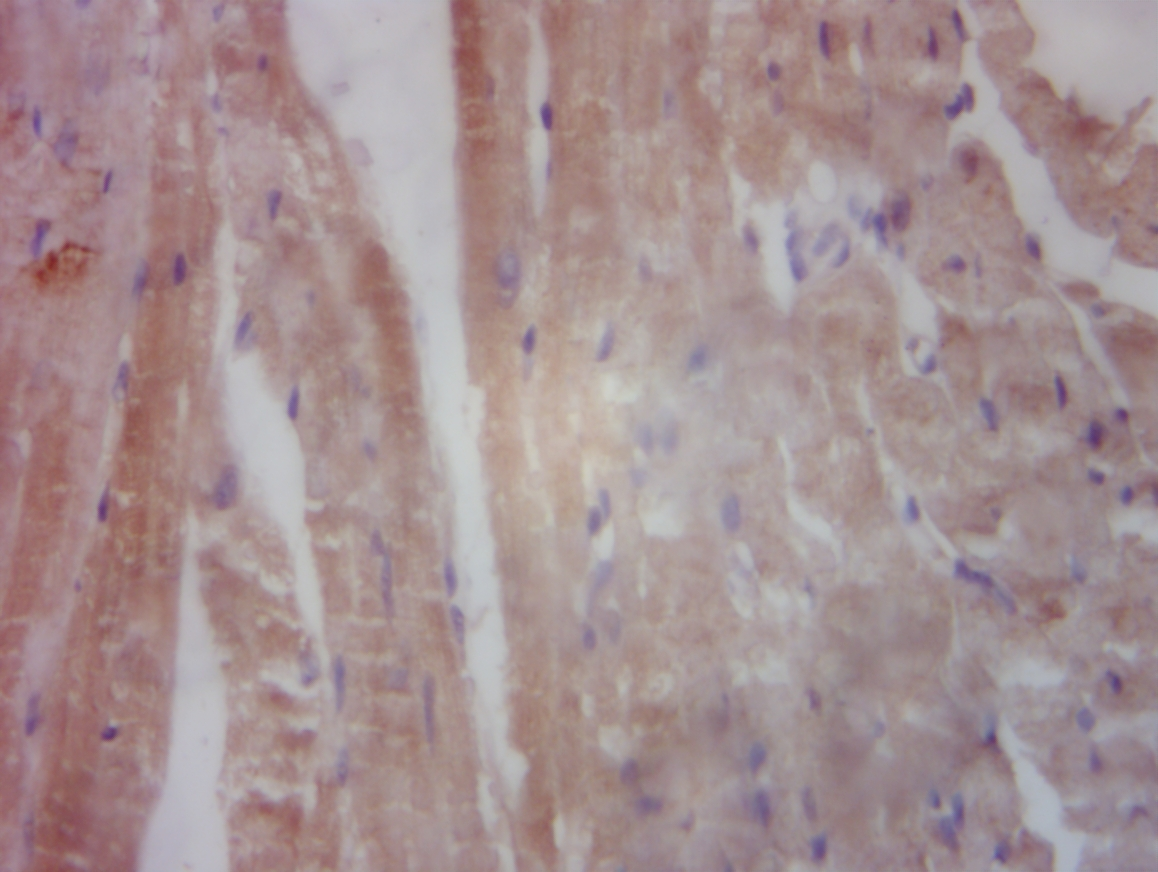


**4-HNE**

**C**

**db/+**

**db/db**

**Ad-NC**

**Ad-NC**

**Ad-sh-ZFAS1**

**Ad-ZFAS1**


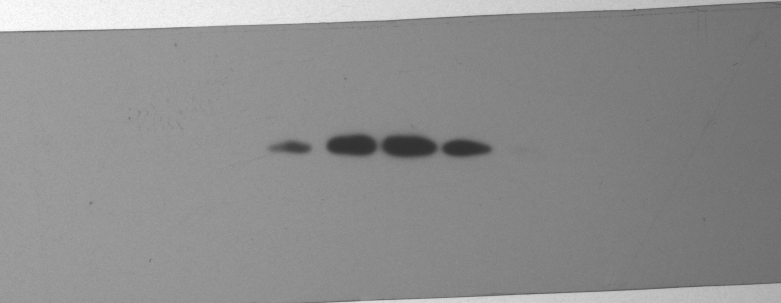


**GPX4**

**
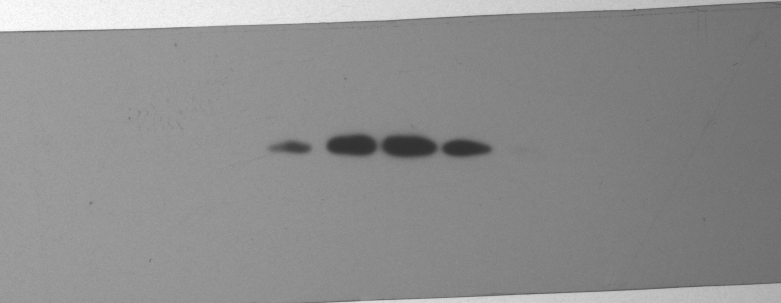
**

**Cleaved caspase 3**

**
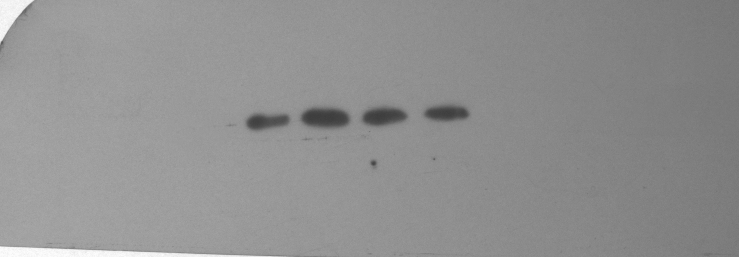
**

**Bax**

**
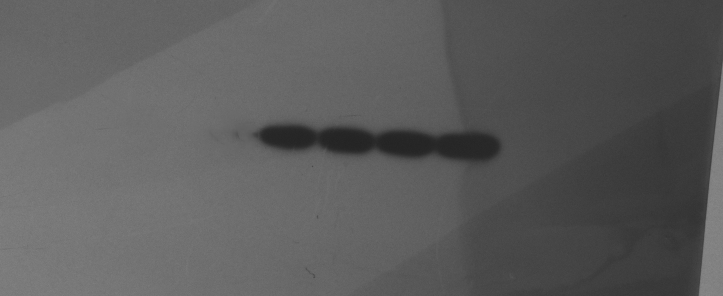
**

**
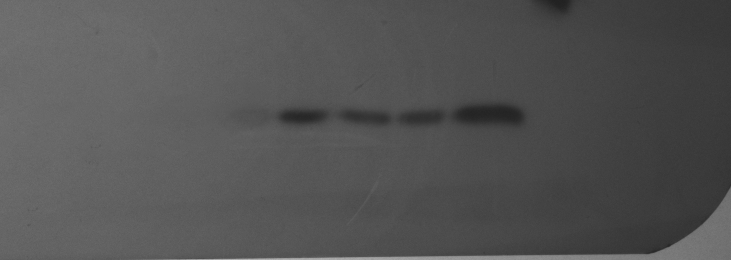
**

**Bcl-2**


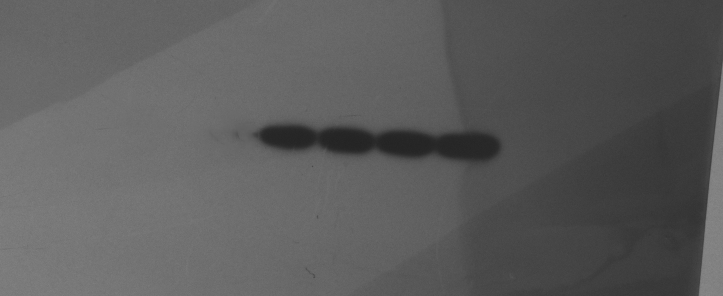


**β-actin**

**E**

**CON+Ad-NC**

**HG+Ad-NC**


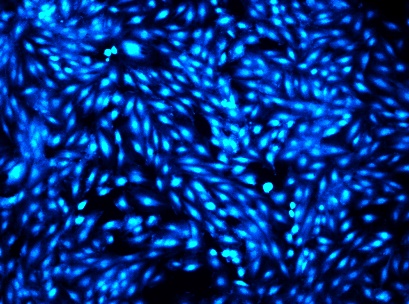


**MBB**


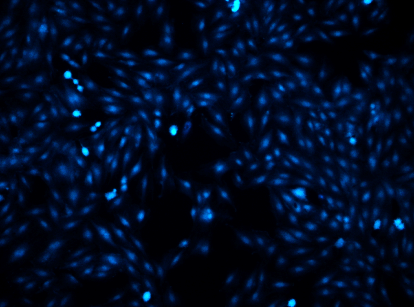


**HG+Ad-ZFAS1**

**HG+Ad-sh-ZFAS1**


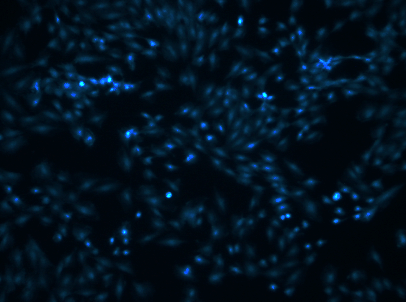

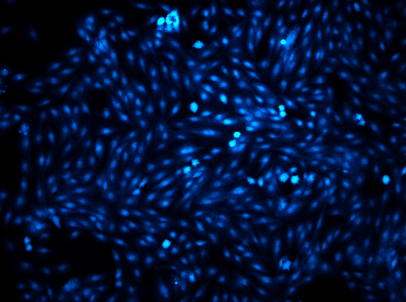


**F**

**CON+Ad-NC**

**HG+Ad-NC**

**HG+Ad-sh-ZFAS1**

**HG+Ad-ZFAS1**


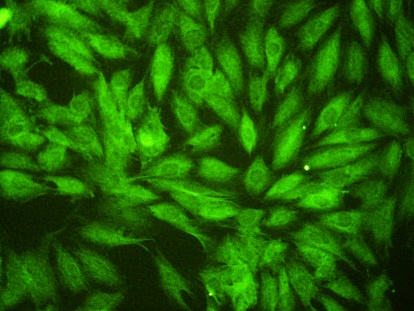


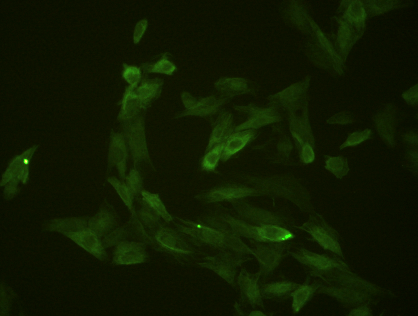

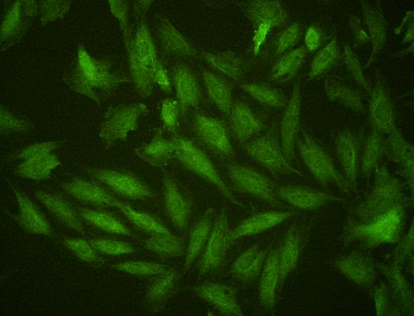

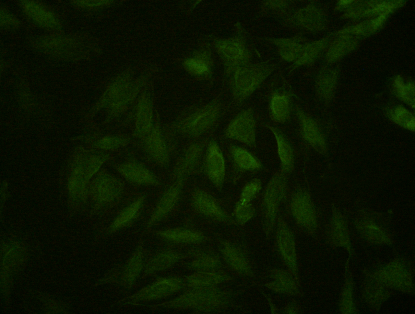


**FTH1**


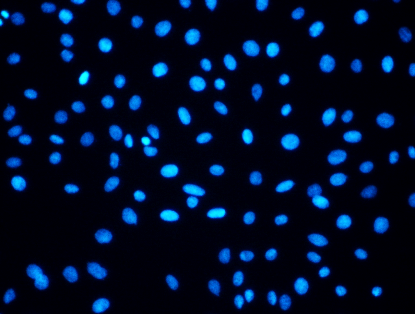


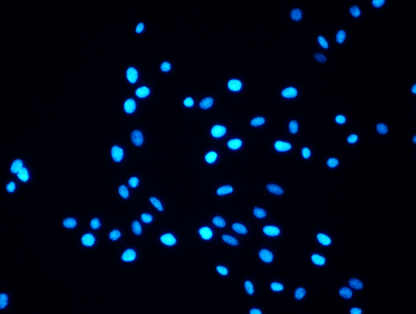

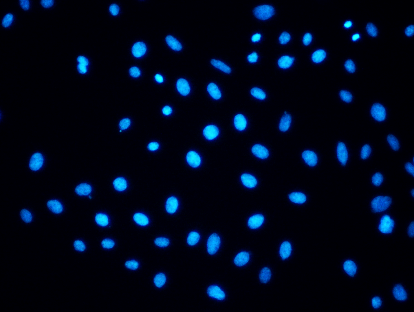

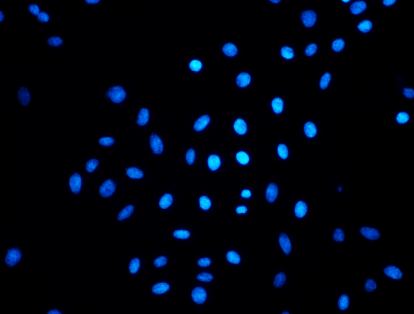


**G**

**HG+Ad-ZFAS1**

**CON+Ad-NC**

**HG+Ad-NC**

**HG+Ad-sh-ZFAS1**


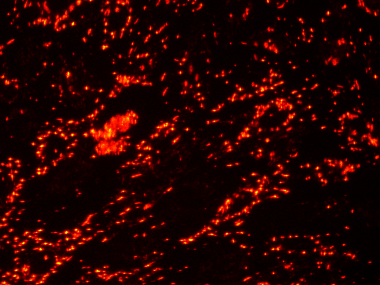


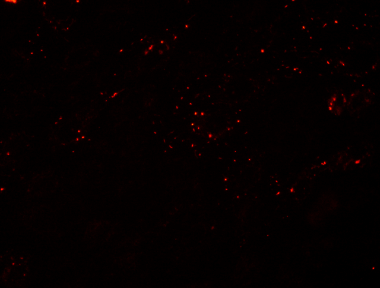

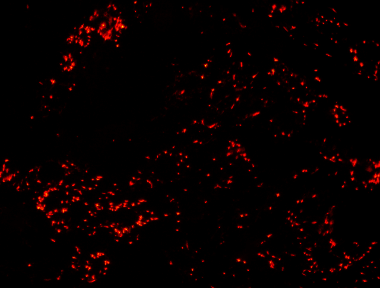

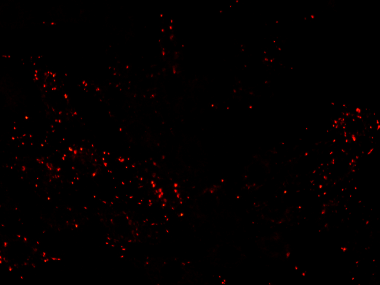


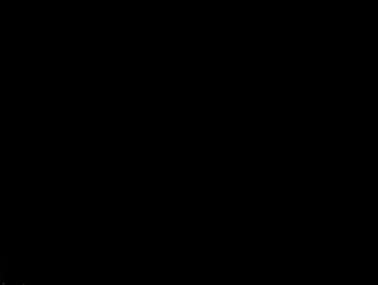


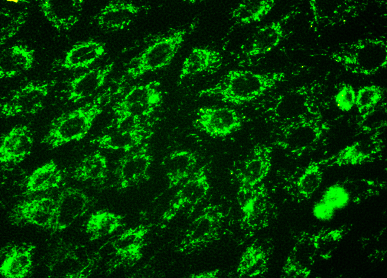

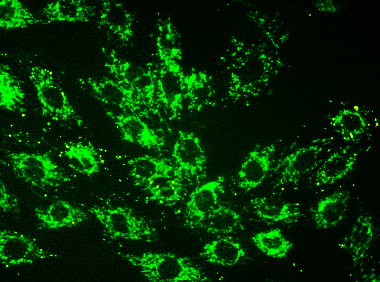

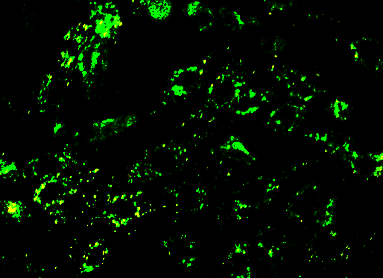


**H**

**HG**

**CON**

**Ad-NC**

**Ad-NC**

**Ad-sh-ZFAS1**

**Ad-ZFAS1**


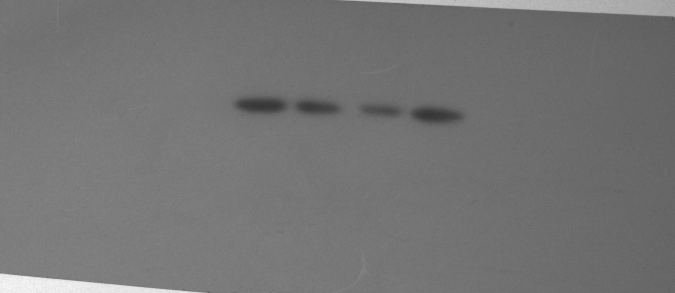


**GPX4**


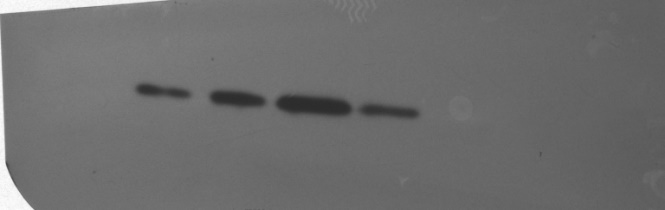


**Cleaved caspase 3**


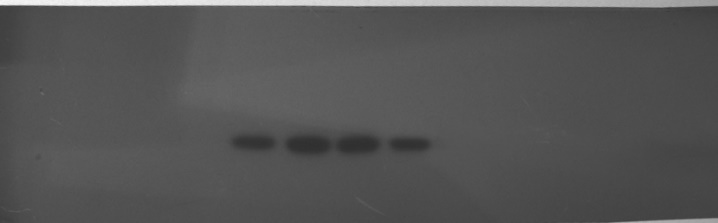


**Bax**


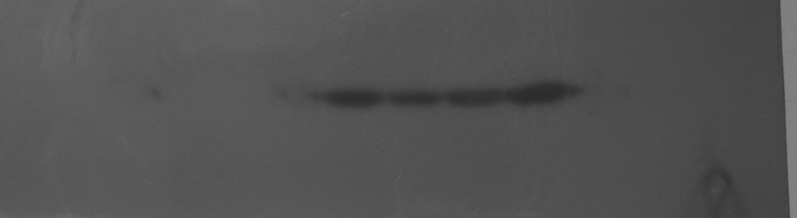


**Bcl-2**


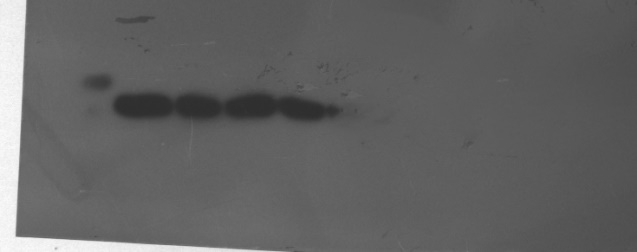


**β-actin**

**Figure 4**

**Random probe**

**G**

**ZFAS1 probe**

**Pull down**

**Input**

**Input**

**Pellet**

**Pellet**


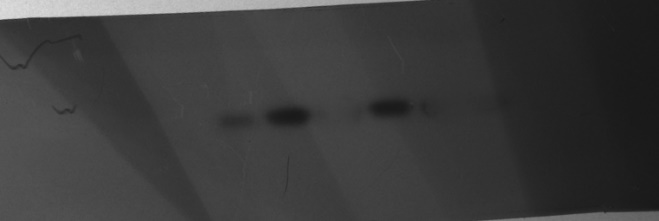


**miR-150-5p**

**U6**


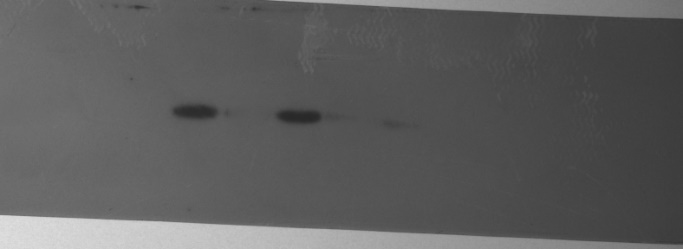


**db/db**

**db/+**

**I**


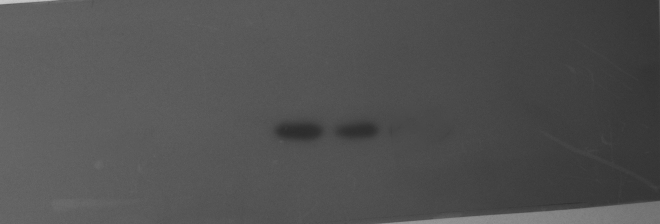


**CCND2**


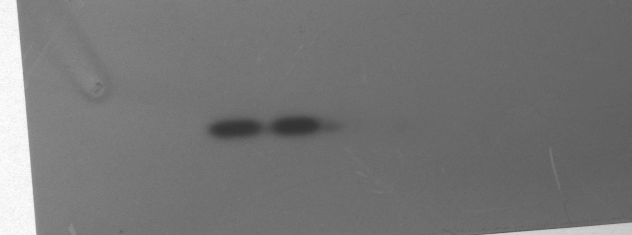


**β-actin**

**HG**

**CON**

**K**

**HG**

**CON**

**CCND2**


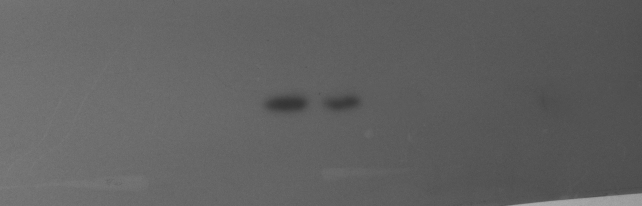


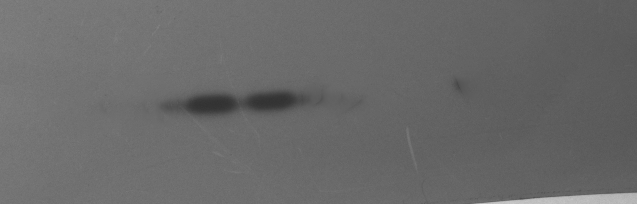


**β-actin**

**Figure 5**

**A**

**db/+ Ad-NC+mimic NC db/db Ad-NC+Mimic NC**


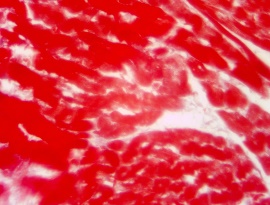

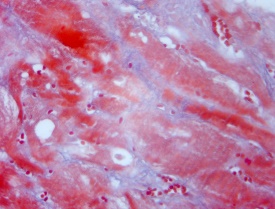


**db/db Ad-sh-ZFAS1+Mimic NC db/db Ad-NC+Mimic miR-150-5p**


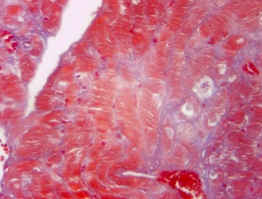

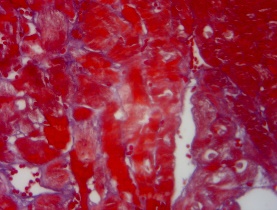


**db/db Ad-ZFAS1+ db/db Ad-sh-ZFAS1+Mimic miR-150-5p**

**Mimic miR-150-5p**


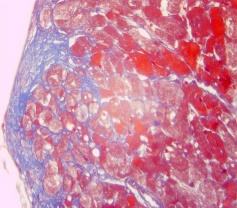

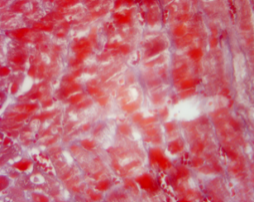


**B**

**db/+ Ad-NC+mimic NC db/db Ad-NC+Mimic NC**


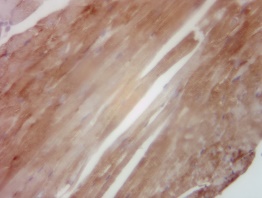


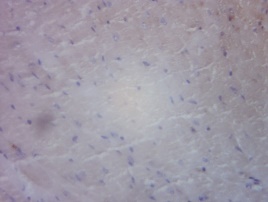


**FTH1**


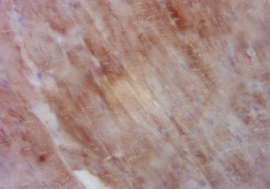

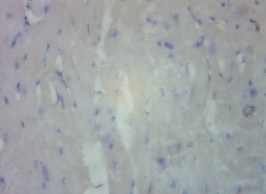


**4-HDY**

**db/db Ad-sh-ZFAS1+Mimic NC db/db Ad-NC+Mimic miR-150-5p**


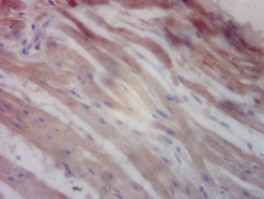


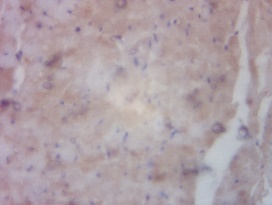


**FTH1**


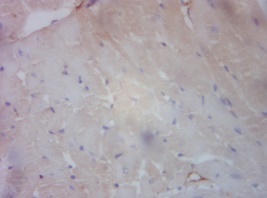


**4-HDY**


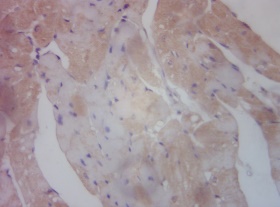


**db/db Ad-ZFAS1+ db/db Ad-sh-ZFAS1+Mimic miR-150-5p**

**Mimic miR-150-5p**


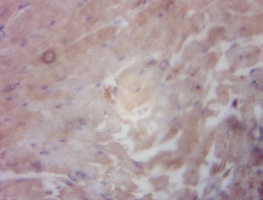

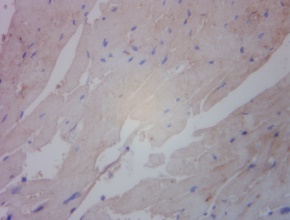


**FTH1**


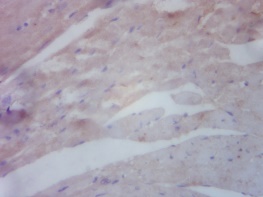

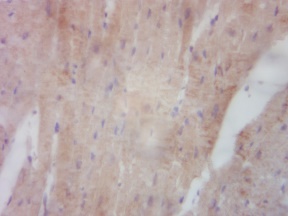


**4-HDY**

**C**

**Ad-NC+**

**Mimic NC**

**Ad-NC**

**+Mimic NC**

**Ad-ZFAS1+**

**Mimic miR-150-5p**

**Ad-sh-ZFAS1**

**+Mimic NC**

**Ad-NC+Mimic miR-150-5p**

**Ad-sh-ZFAS1+Mimic miR-150-5p**

**HG**

**CON**


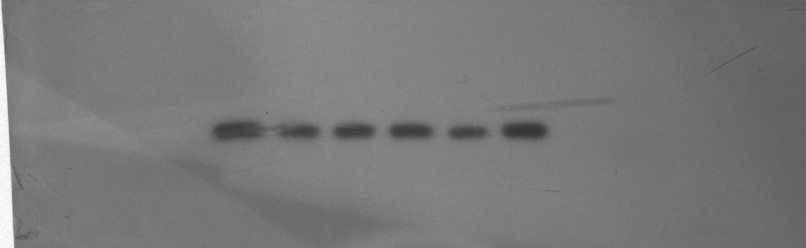


**CCND2**


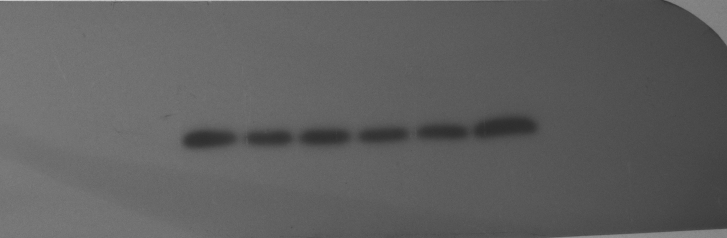


**GPX4**


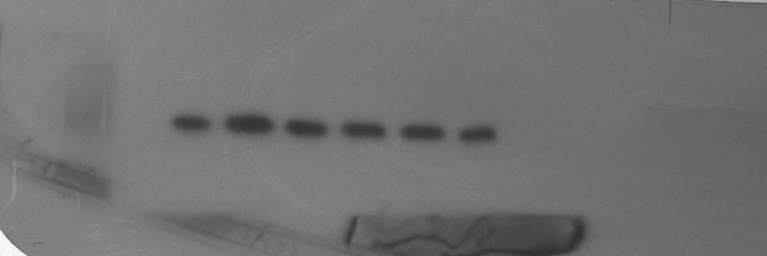


**Cleaved caspase 3**


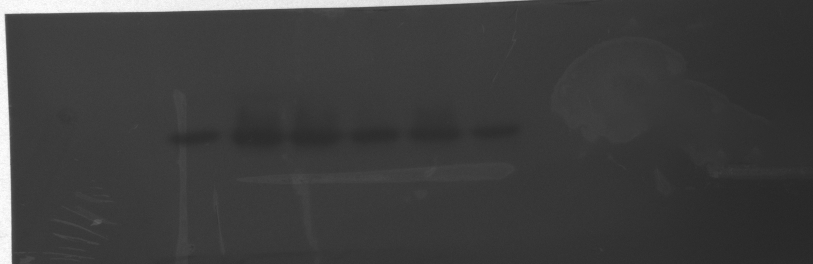


**Bax**


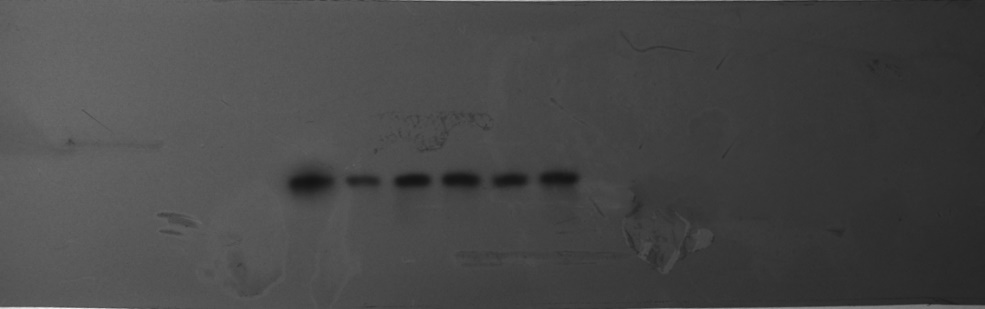


**Bcl-2**


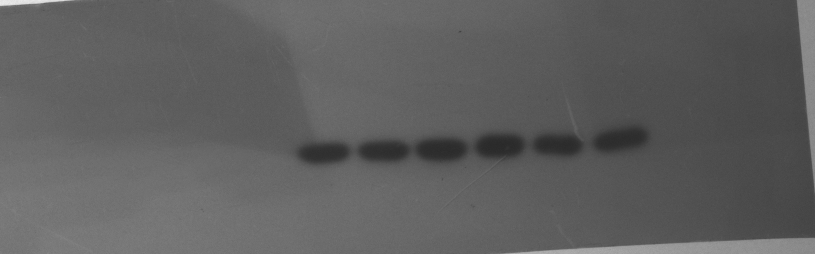


**β-actin**

**E**

**CON +Ad-NC+mimic NC HG+Ad-NC+Mimic NC**


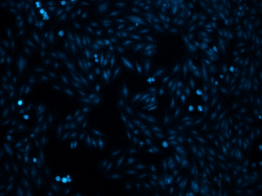

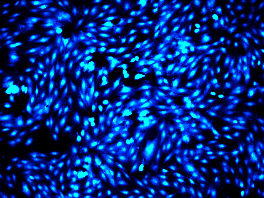


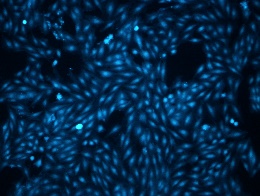
 **HG+Ad-ZFAS1+ Mimic NC HG+ Ad-NC+Mimic miR-150-5p**


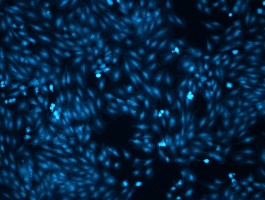


**HG+ Ad-ZFAS1+ HG+Ad-sh-ZFAS1+Mimic miR-150-5p**

**Mimic miR-150-5p**


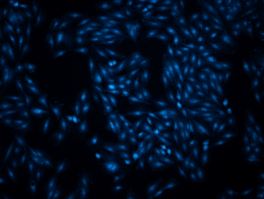


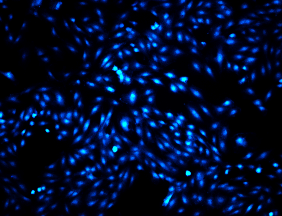


**F**


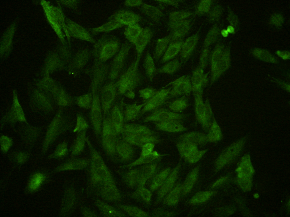

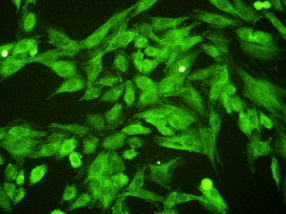
**CON+ Ad-NC+mimic NC HG+ Ad-NC+Mimic NC**


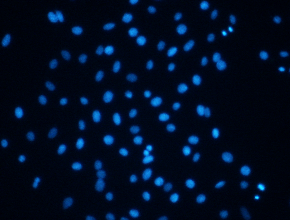

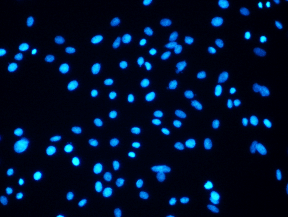


**HG+ Ad-sh-ZFAS1+Mimic NC HG+ Ad-NC+Mimic miR-150-5p**


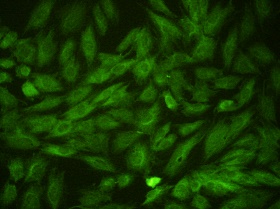


**HG+ Ad-ZFAS1+ HG+Ad-sh-ZFAS1+Mimic miR-150-5p**

**Mimic miR-150-5p**

**G**

**CON+ Ad-NC+mimic NC HG+ Ad-NC+Mimic NC**

**HG+ Ad-sh-ZFAS1+Mimic NC HG+ Ad-NC+Mimic miR-150-5p**

**HG+ Ad-ZFAS1+ HG+Ad-sh-ZFAS1+Mimic miR-150-5p**

**Mimic miR-150-5p**

**H**

**CCND2**

**GPX4**

**Cleaved caspase 3**

**Bcl-2**

**Bax**

**β-actin**

**Figure 6**

**A**

**db/+ Ad-NC db/db+ Ad-NC**

**db/db+ Ad-sh-ZFAS1 db/db+ Ad-CCND2**

**db/db+ db/db+**

**Ad-ZFAS1+ Ad-sh-ZFAS1+**

**Ad-sh-CCND2 Ad-CCND2**

**B**

**db/+ Ad-NC db/db+ Ad-NC**

**FTH1**

**4-HDY**

**db/db+ Ad-sh-ZFAS1 db/db+ Ad-CCND2**

**FTH1**

**4-HDY**

**db/db+ db/db+**

**Ad-ZFAS1+ Ad-sh-ZFAS1+**

**Ad-sh-CCND2 Ad-CCND2**

**FTH1**

**4-HDY**

**C**

**Bax**

**Bcl-2**

**GPX4**

**Cleaved caspase 3**

**β-actin**

**E**

**E**

**CON +Ad-NC HG+Ad-NC**

**HG+Ad-sh-ZFAS1 HG+Ad-CCND2**

**HG+Ad-ZFAS1+ HG+Ad-sh-ZFAS1+Ad-sh-CCND2**

**Ad-CCND2**

**F**

**CON +Ad-NC HG+Ad-NC**

**HG+Ad-sh-ZFAS1 HG+Ad-CCND2**

**HG+Ad-ZFAS1+ HG+Ad-sh-ZFAS1+Ad-sh-CCND2**

**Ad-CCND2**

**G**

**CON +Ad-NC HG+Ad-NC**

**HG+Ad-sh-ZFAS1 HG+Ad-CCND2**

**HG+Ad-ZFAS1+ HG+Ad-sh-ZFAS1+Ad-sh-CCND2**

**Ad-CCND2**

**H**

**GPX4**

**Cleaved caspase 3**

**Bax**

**Bcl-2**

**β-actin**
